# In Situ Remodeling of Efferocytosis via Lesion‐Localized Microspheres to Reverse Cartilage Senescence

**DOI:** 10.1002/advs.202400345

**Published:** 2024-03-13

**Authors:** Wei Xiong, Zeyu Han, Sheng‐Long Ding, Haoran Wang, Yawei Du, Wenguo Cui, Ming‐Zhu Zhang

**Affiliations:** ^1^ Department of Foot and Ankle Surgery Beijing Tongren Hospital Capital Medical University Beijing 100730 P. R. China; ^2^ Department of Orthopaedics Shanghai Key Laboratory for Prevention and Treatment of Bone and Joint Diseases Shanghai Institute of Traumatology and Orthopaedics Ruijin Hospital Shanghai Jiao Tong University School of Medicine 197 Ruijin 2nd Road Shanghai 200025 P. R. China

**Keywords:** cartilage senescence, efferocytosis, Hydrogel microspheres, lesion‐localized, osteoarthritis

## Abstract

Efferocytosis, an intrinsic regulatory mechanism to eliminate apoptotic cells, will be suppressed due to the delayed apoptosis process in aging‐related diseases, such as osteoarthritis (OA). In this study, cartilage lesion‐localized hydrogel microspheres are developed to remodel the in situ efferocytosis to reverse cartilage senescence and recruit endogenous stem cells to accelerate cartilage repair. Specifically, aldehyde‐ and methacrylic anhydride (MA)‐modified hyaluronic acid hydrogel microspheres (AHM), loaded with pro‐apoptotic liposomes (liposomes encapsulating ABT263, A‐Lipo) and PDGF‐BB, namely A‐Lipo/PAHM, are prepared by microfluidic and photo‐cross‐linking techniques. By a degraded porcine cartilage explant OA model, the in situ cartilage lesion location experiment illustrated that aldehyde‐functionalized microspheres promote affinity for degraded cartilage. In vitro data showed that A‐Lipo induced apoptosis of senescent chondrocytes (Sn‐chondrocytes), which can then be phagocytosed by the efferocytosis of macrophages, and remodeling efferocytosis facilitated the protection of normal chondrocytes and maintained the chondrogenic differentiation capacity of MSCs. In vivo experiments confirmed that hydrogel microspheres localized to cartilage lesion reversed cartilage senescence and promoted cartilage repair in OA. It is believed this in situ efferocytosis remodeling strategy can be of great significance for tissue regeneration in aging‐related diseases.

## Graphical abstract

This work introduced the innovative A‐Lipo/PAHM microsphere, localizing to degraded cartilage. The released A‐Lipo is internalized by the Sn‐chondrocytes, subsequently, the apoptotic Sn‐chondrocytes are eliminated via efferocytosis. Furthermore, recruited stem cells played a role in promoting the regeneration of degraded cartilage. This strategy serves as a promising approach for reversing cartilage senescence.

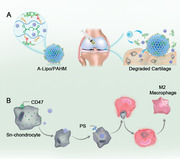

## 1 Introduction

Efferocytosis is a process by which phagocytes (e.g., macrophages, dendritic cells) eliminate the apoptotic cells undergone programmed death.^[^ ^1^ ^]^ With the deep‐going research, there is growing evidence that efferocytosis has decent immunomodulatory effects, which play a crucial role in tissue regeneration. Efferocytosis not only avoids tissue immunoreaction of accumulated uncleared apoptotic cells but also participates in the conversion of macrophages into a proliferative phenotype to promote tissue repair.^[^ ^1^, ^2^ ^]^ However, in some aging‐related diseases, efferocytosis is suppressed owing to the delayed programmed apoptosis of senescent cells that cannot be eliminated timely, forming “zombie” cells.^[^ ^3^ ^]^ Moreover, the excreted senescence‐associated secretory phenotype (SASP) impairs the organism's function and is detrimental to tissue regeneration by stem cells.^[^ ^4^ ^]^ It becomes imperative to remodel macrophage efferocytosis by promoting apoptosis of senescent cells, especially in the aging‐related lesion demanding tissue regeneration.

Osteoarthritis (OA) is a prevalent senile disease that features the degeneration of cartilage, the alteration of subchondral bone, osteophyte formation, and inflammation in synovial.^[^ ^5^ ^]^ Notably, cellular senescence represents a pivotal contributor to OA pathology.^[^ ^6^ ^]^ In the healthy articular cartilage, chondrocytes are almost the only cell types, and their secretion of the extracellular matrix (ECM), primarily composed of type II collagen and glycosaminoglycans, maintains the normal structure and mechanical properties of articular cartilage.^[^ ^7^, ^8^ ^]^ Some pathogenic factors, such as oxidative stress and abnormal mechanical loads, contribute to chondrocyte senescence.^[^ ^8^, ^9^ ^]^ What was worse, Sn‐chondrocytes would excrete SASPs (e.g., IL‐6, MMPs) to induce further cellular senescence in joints, even other cell types in the adjacent microenvironment.^[^ ^8^, ^10^ ^]^ This uncontrollable aging phenotype severely disrupts the balance between the synthesis and degradation of the cartilage ECM, ultimately creating an unfavorable environment for cartilage regeneration. Currently, therapeutic agents targeting Sn‐chondrocytes in OA could be mainly divided into senomorphics and senolytics.^[^ ^6b,c^ ^]^ However, senomorphics could not essentially alleviate all tissue damages caused by various SASPs.^[^ ^6b,c^ ^]^ Similarly, although senolytics could induce apoptosis of Sn‐chondrocytes theoretically, clinical studies did not support their anticipated efficacy in trials of larger sample sizes.^[^ ^11^ ^]^ In essence, the previous researches overlook the significance of efferocytosis — a process essential for the clearance of occupying Sn‐chondrocytes and establishing an anti‐inflammatory microenvironment conducive to cartilage regeneration.^[^ ^12^ ^]^ Thus, we believe that inducing an efficient in situ efferocytosis of macrophages to eliminate “zombie” Sn‐chondrocytes represents a promising approach to reverse cartilage senescence and re‐establish tissue homeostasis.

In the tissue regeneration field, including cartilage repair, the scaffold is a crucial component providing the mimic ECM to support stem cells for proliferation and differentiation.^[^ ^13^ ^]^ Designing proper tissue‐engineered scaffolds based on cartilage‐specific structural properties would significantly promote cartilage regeneration for OA treatment.^[^ ^14^ ^]^ In our previous studies, we created polydopamine‐coated and cationized HAMA microspheres to obtain the cartilage adhesion property through hydrogen bonds or charge interactions, which could enhance the lubricating ability to alleviate OA.^[^ ^15^ ^]^ While this non‐specific cartilage adhesion noticeably improves joint wear, it represents a palliative approach and fails to facilitate the regeneration of damaged cartilage. Moreover, the ECM around damaged cartilage still undergoes extensive degradation.^[^ ^8a^ ^]^ Hence, there arises a crucial need for precise lesion targeting and regeneration promotion. Studies have indicated that the degradation of cartilage ECM exposes numerous amino groups due to depolymerization of the triple helical structure of type II collagen, the characteristic of which has been participated in specific targeting.^[^ ^16^ ^]^ The lesion‐located aldehyde‐modified microspheres could serve as potent tools as tissue‐engineered scaffolds and drug carriers. The in situ adhered porous microspheres would provide mimic ECM for tissue regeneration. Anti‐aging agents, such as senolytics, or stem cell recruitment factors could be applied for lesion precise delivery for further in situ functionalization. Therefore, based on the precise lesion adhesion, the aldehyde‐modified microspheres could afford a decent in situ microenvironment for cartilage repair.

**Scheme 1:**
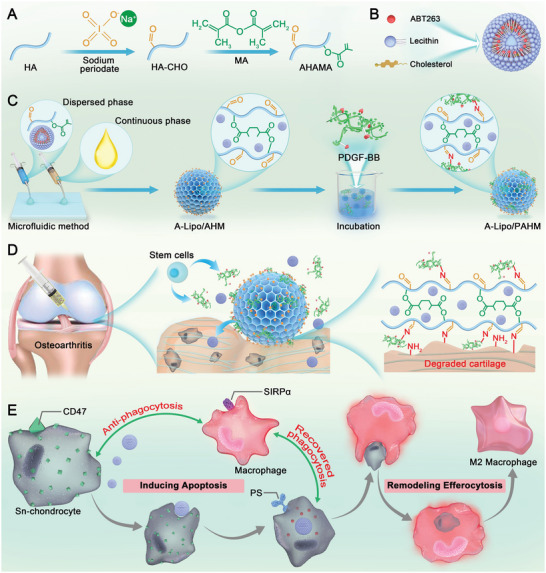
Design and preparation of A‐Lipo/PAHM and the mechanism of reversal of cartilage senescence via efferocytosis by A‐Lipo/PAHM. A) Synthesis of AHAMA hydrogels by sodium periodate oxidation and MA modification. Hyaluronic acid (HA), methacrylic anhydride (MA). B) Preparation of A‐Lipo. C) Fabrication of A‐Lipo/PAHM via microfluidic methods. D) The treatment of OA with intra‐articular injection of A‐Lipo/PAHM. Degraded cartilage in OA exposes numerous amino groups, and aldehyde‐modified A‐Lipo/PAHM can localize to the degraded cartilage through the Schiff base reaction. The released PDGF‐BB recruits endogenous stem cells to repair the damaged cartilage. E) Mechanism of reversing cartilage senescence via remodeling efferocytosis by A‐Lipo/PAHM. CD47, a “don't eat‐me” signal, is overexpressed in senescent cells, which inhibits macrophage phagocytosis of senescent cells via the CD47‐SIRPα axis. Phosphatidylserine (PS), an “eat‐me” signal, is exposed on the apoptotic cell membrane. Released A‐Lipo promoted the apoptosis of Sn‐chondrocytes, and the apoptotic Sn‐chondrocytes were phagocytosed by macrophage efferocytosis. Macrophages then switched to the anti‐inflammatory M2 phenotype.

Currently, the pro‐apoptosis strategy of senescent cells is generally acknowledged for aging‐related disease therapy. However, the further efferocytosis effect and the secondary alteration of tissue homeostasis are always ignored, especially in the in situ degenerative lesion. In this study, we unveil a novel approach for in situ efferocytosis remodeling, achieved through engineered lesion‐adherent microspheres, to reverse cartilage senescence and reinstate articular cartilage tissue homeostasis (**Scheme** **1**). Herein, AHM were prepared using aldehyde‐ and methacrylic anhydride (MA)‐modified hyaluronic acid hydrogels (AHAMA) via microfluidic and photo‐cross‐linking techniques. Meanwhile, A‐Lipo, a pro‐apoptotic agent, and a stem cells recruitment factor, PDGF‐BB, were loaded onto AHM, respectively, to fabricate A‐Lipo/PAHM. After intra‐articular injections, A‐Lipo/PAHM localized to the degraded cartilage surface through Schiff base reactions, providing the in situ mimic ECM. PDGF‐BB and A‐Lipo were then released to recruit the surrounding stem cells and hunt Sn‐chondrocytes, respectively. In situ efferocytosis would be activated due to the emerged apoptotic signals to remove the occupying Sn‐chondrocytes and facilitate anti‐inflammatory immune regulation simultaneously. The recruited stem cells would be promoted for enhanced cartilage regeneration accordingly. In vitro and in vivo data validated the results, including in situ adhesion of microspheres, senescence reversal activity, efferocytosis remodeling, and OA efficacy. Together, we developed lesion‐localized hydrogel microspheres that remodel efferocytosis in situ to reverse cartilage senescence, which effectively alleviates OA, providing a novel approach to treating other aging‐related diseases.

## 2 Results and Discussion

### 2.1 Preparation and Characterization of A‐Lipo/PAHM

**Figure 1:**
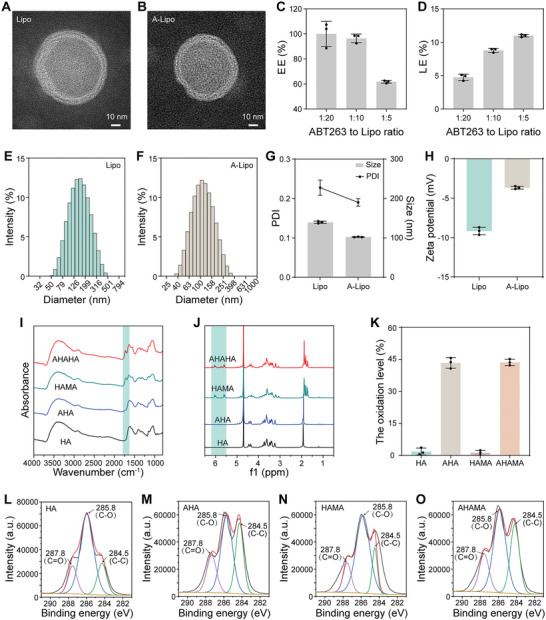
Characterization of liposomes and AHAMA hydrogels. A,B) TEM images of Lipo and A‐Lipo. Scale bar: 10 nm. C,D) Encapsulation efficiency (EE) and loading efficiency (LE) of ABT263. Data are presented as mean ± SD (*n* = 3). E,F) Evaluation of hydrodynamic diameter distribution of Lipo and A‐Lipo using Malvern‐ Zetasizer. G,H) Size, PDI, zeta potential of Lipo and A‐Lipo. Data are presented as mean ± SD (*n* = 3). I) HA, AHA, HAMA, and AHAMA Hydrogel FTIR spectra. J) HA, AHA, HAMA, and AHAMA Hydrogel ^1^H‐NMR spectra in D_2_O (600 MHz). K) Quantification of aldehyde oxidation levels of AHA and AHAMA hydrogels using hydroxylamine hydrochloride titration analysis. Data are presented as mean ± SD (*n* = 3). L–O) Hydrogel C(1s) XPS regions of HA, AHA, HAMA, and AHAMA.

First, the thin film dispersion technique was used to fabricate liposomes composed of cholesterol, lecithin, and ABT263. Transmission electron microscopy (TEM) images indicated that both the liposome (Lipo) and A‐Lipo exhibited the membrane structure of a spherical lipid‐like bilayer (**Figure** **1**A,B). We then investigated the encapsulation efficiency (EE) and drug loading efficiency (LE) of ABT263 within the Lipo. The EE of ABT263 was up to 96.5% and the LE was ≈8.8% at a 1:10 mass ratio of ABT263 to Lipo (Figure 1C,D). This liposomal formulation of A‐Lipo was used for subsequent experiments. The particle size distributions of Lipo and A‐Lipo were examined using dynamic light scattering (DLS) (Figure 1E,F). The average sizes of Lipo and A‐Lipo were 139.7 ± 2.7 and 102.3 ± 0.7 nm, respectively. The average PDI of Lipo and A‐Lipo were 0.23 ± 0.02 and 0.19 ± 0.01, which indicated that Lipo and A‐Lipo were well dispersed (Figure 1G). Furthermore, the zeta potentials of Lipo and A‐Lipo were determined using DLS, yielding values of −9.2 ± 0.5 and −3.7 ± 0.2 mV, respectively (Figure 1H). Cartilage tissue is negatively charged and has a very dense ECM. The particle size and charge significantly affect their ability to penetrate cartilage, thereby influencing their therapeutic efficacy.^[^ ^17^ ^]^ No discernible changes were found in the size or PDI of Lipo and A‐Lipo over 28 days, suggesting that the liposomes remained stable over a long period of time (Figures S1 and S2, Supporting Information). Moreover, the release of ABT263 from A‐Lipo further indicated that the liposomes maintained stability over time (Figure S3, Supporting Information). Liposomes featuring a bilayer membrane structure with a hydrophilic core suitable for loading hydrophilic drugs and a hydrophobic lipid bilayer membrane for encapsulating hydrophobic drugs are known for their biocompatibility, controlled drug release, and potential for targeted drug delivery, making them extensively used in biomedical applications.^[^ ^18^ ^]^

Next, we modified hyaluronic acid (HA) with an aldehyde group and methacrylic anhydride (MA) to obtain AHAMA hydrogels. The Fourier transform infrared spectroscopy (FTIR) depicted a distinctive C═C stretching band at 1700 cm^−1^ in both AHAMA and HAMA hydrogels (Figure 1I). Two new peaks corresponding to the protons of the C═C bond of MA were observed in the ^1^H NMR spectrum at 5.7 and 6.1 ppm (Figure 1J). The MA grafting rates of AHAMA and HAMA, determined by the ratio of the integral area of the methyl proton peak of MA to the methyl proton peak of HA or AHA,^[^ ^19^ ^]^ were 56.3% and 63.9%, respectively, which were calculated using the ^1^H NMR spectra results (Figures S4 and S5, Supporting Information). The oxidation levels of AHA and AHAMA were then determined by hydroxylamine hydrochloride titration. The aldehyde contents of AHA and AHAMA were 43.3% and 43.6%, respectively (Figure 1K). XPS results showed that the C═O peak area of AHA was greater than that of HA (Figure 1L,M). Similarly, the C═O peak area of AHAMA was larger than that of HAMA (Figure 1N,O). Conversely, the C═O peak area of AHA was not obviously changed compared with that of AHAMA (Figure 1M,O). These results confirmed the presence of aldehyde groups in AHA and AHAMA. Polysaccharides oxidized by sodium periodate, termed as aldehyde polysaccharides, have low toxicity, are biocompatible and biodegradable, which gain a wide range of applications.^[^ ^20^ ^]^ HA enhances joint lubrication by intra‐articular injection,^[^ ^21^ ^]^ and the HAMA hydrogel contributes to joint lubrication and serves as a suitable vehicle for drug delivery.^[^ ^22^ ^]^ Compared with the HAMA hydrogel, the AHAMA hydrogel featuring an aldehyde group exhibit a tissue adhesion capability and demonstrates a superior cartilage repair potential.^[^ ^16^, ^23^ ^]^

**Figure 2:**
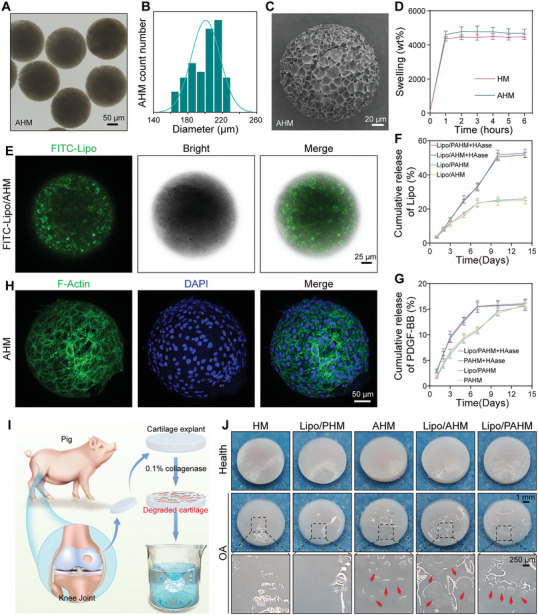
Characterization of lesion‐localized hydrogel microspheres. A) Images of AHM acquired by the bright‐field microscope. Scale bar: 50 µm. B) Diameter distribution of AHM calculated using imageJ. C) Representative SEM images of AHM. Scale bar: 20 µm. D) Swelling rate of HM and AHM. Data are presented as mean ± SD (*n* = 6). E) LSCM images of FITC‐Lipo/AHM. FITC‐Lipo (Green), AHM (Bright). Scale bar: 25 µm. F) Measurement of the cumulative release rate of Lipo in Lipo/AHM and Lipo/PAHM under PBS or PBS containing HAase (1500 U mL^−1^) by quantification of the fluorescence intensity of FITC‐labeled Lipo. Data are presented as mean ± SD (*n* = 3). G) Measurement of the cumulative release rate of PDGF‐BB in PAHM and Lipo/PAHM under PBS or PBS containing HAase (1500 U mL^−1^) by using ELISA kit. Data are presented as mean ± SD (*n* = 3). H) Representative fluorescence images of BMSCs implanted on AHM. F‐Actin (Green), DAPI (Blue). Scale bar: 50 µm. I) Scheme of microsphere localization experiment for degraded porcine cartilage. J) The digital photographs of HM, A‐Lipo/PHM, AHM, A‐Lipo/AHM, and A‐Lipo/PAHM adhered to the surface of healthy or OA cartilage explants. The red arrows indicate cartilage‐adhered microspheres. Scale bar: 1 mm (above); 250 µm (below).

Last, A‐Lipo/PAHM was prepared using microfluidic and photo‐cross‐linking techniques. Representative images from optical microscopy showed that AHM and HM were spherical and structurally intact (**Figure** **2**A; Figure S6A, Supporting Information). AHM and HM exhibited a narrow particle size distribution. The particle size of AHM was 200.6 ± 16.6 µm, and that of HM was 188.8 ± 13.2 µm, which were relatively close to each other (Figure 2B; Figure S6B, Supporting Information). The size of hydrogel microspheres could be adjusted by controlling the velocity of the dispersed and continuous phases in microfluidics.^[^ ^24^ ^]^ The scanning electron microscope (SEM) images of AHM and HM presented its porous structure (Figure 2C; Figure S7, Supporting Information). As shown in Figure 2D, AHM and HM reached the swelling equilibrium within 1 h, exhibiting their good water absorption and swelling properties. The degradation ability of AHM and HM was investigated in PBS containing hyaluronidase in vitro. Light microscopy revealed slight degradation of AHM at day 14, and at day 42, AHM degradation was very pronounced (Figure S8, Supporting Information). However, the spherical structure of HM remained relatively intact with insignificant degradation over the same time. The degradability of bioactive materials is an essential parameter for their ability to be transplanted in vivo. We found that the aldehyde group modification improved the degradation properties of HAMA. Thus, compared with HM, AHM may have better application prospects in tissue regeneration. FITC was employed to label Lipo for applying to evaluate the construction of the micro‐nanostructure consisting of liposomes and microspheres. Laser confocal microscopy revealed the presence of FITC‐labeled liposomes in microspheres (Figure 2E). The zeta potential of HM, AHM, A‐Lipo/HM, A‐Lipo/AHM, A‐Lipo/PHM, and A‐Lipo/PAHM was measured by DLS (Figure S9, Supporting Information). All types of microspheres were negatively charged. Aldehyde modification and the addition of liposomes decreased the zeta potential of the microspheres, whereas the incorporation of PDGF‐BB increased the zeta potential of the microspheres. This may be caused by the feature that liposomes are normally negatively charged and PDGF‐BB is positively charged.^[^ ^18^, ^25^ ^]^ Exogenous stem cell transplantation may have shortcomings such as a low survival rate, immunogenicity, restricted cell source, and infection risk.^[^ ^26^ ^]^ The recruitment of endogenous stem cells to regenerate damaged tissues is currently receiving considerable attention in tissue regeneration.^[^ ^27^ ^]^ Multiple types of MSCs, such as bone marrow‐resident MSCs, synovial‐resident MSCs, synovial fluid‐resident MSCs, and so on, are abundant in the joint cavity. In OA, a combined strategy of resident MSCs, tissue‐engineered scaffolds and recruitment of resident MSCs can significantly improve cartilage repair.^[^ ^28^ ^]^ PDGF‐BB can recruit endogenous MSCs, but its short half‐life limits its efficacy in vivo.^[^ ^25^, ^29^ ^]^ The sustained release of PDGF‐BB from hydrogel microspheres could enhance its ability to recruit stem cells. PDGF‐BB is positively charged and its amino group can undergo a Schiff base reaction with the aldehyde group, therefore, negatively charged and aldehyde‐modified AHM can load PDGF‐BB via electrostatic interaction and Schiff base reaction. The PDGF‐BB loading efficiency of both AHM and HM reached ≈95% (Figures S10 and S11, Supporting Information). We further investigated the behavior of microsphere delivery systems for controlled release of Lipo or PDGF‐BB in vitro (Figure 2F,G; Figures S12 and S13, Supporting Information). In PBS containing hyaluronidase, the cumulative release of Lipo reached ≈50% in AHM or HM at day 14. Conversely, the cumulative release rate of Lipo in PBS reached only half of that in PBS containing hyaluronidase. PDGF‐BB loading did not affect the release behavior of Lipo. As the hydrogel microspheres degrade, Lipo can be released from the cross‐linked network. The cumulative release of PDGF‐BB reached ≈15% at day 14 for both AHM and HM. Similarly, Lipo‐loaded microspheres did not affect the release of PDGF‐BB. To verify the tissue adhesion ability of aldehyde‐modified microspheres, we evaluated the adhesion of microspheres with cells or degraded cartilage. Representative fluorescence microscopy images showed little BMSCs attached to HM, whereas Calcein/AM‐stained BMSCs were visible in AHM, A‐Lipo/AHM, and A‐Lipo/PAHM (Figure S14, Supporting Information). Laser confocal microscopy images showed a large number of BMSCs growing uniformly on the AHM surface (Figure 2H). To assess the cartilage lesion localized ability, healthy porcine cartilage explants treated with 0.5% collagenase was used to mimic degraded cartilage in OA (Figure 2I). The photographs in Figure 2J show that neither AHM nor HM adhered to the surface of healthy porcine cartilage. However, adherent AHM, A‐Lipo/AHM, and A‐Lipo/PAHM were all observed on the surface of OA porcine cartilage.

Hydrogel microspheres are an effective drug delivery system due to their biocompatibility, injectability, and ease of modification.^[^ ^30^ ^]^ To enhance the loading efficiency of hydrophobic drugs, micro‐nanostructure delivery systems were chosen for investigation.^[^ ^31^ ^]^ We have developed various functionalized HAMA hydrogel microspheres, which can be used as drug delivery systems, have excellent lubricating properties, and are widely used in OA studies.^[^ ^15^, ^32^ ^]^ Undoubtedly, precise recognition of damaged cartilage is more conducive to relieving OA. The ECM of degraded cartilage exposes a large number of amino groups that can be localized by the Schiff base reaction between the aldehyde group of AHM and the amino group on the degraded cartilage surface.

### 2.2 Construction of a Dox‐Induced Chondrocyte Senescence Model In Vitro

Senescent cells exhibit some common features such as increased SA‐β‐Gal activity, higher expression of p53, p16^INK4a^, or p21 (cell cycle protein‐dependent kinase inhibitors), and a strong SASP.^[^ ^8^, ^33^ ^]^ In the previous studies, ionizing irradiation, Doxorubicin (DOX), and hydrogen peroxide have been widely used to induce chondrocyte senescence.^[^ ^12^, ^34^ ^]^ DOX, a DNA‐damaging chemical agent, will lead to cell cycle arrest, DNA damage, telomere shortening, and increased expression of p16^INK4a^ at a low dose, which is widely used to induce cell senescence in vitro.^[^ ^35^ ^]^ In this study, we used DOX to induce chondrocyte senescence in vitro, and its senescent phenotype was identified. Compared to the control group, optical microscopy revealed obvious blue staining in the Dox group, and its SA‐β‐Gal‐positive cells ratio was calculated to be 76% (Figure S15, Supporting Information). We then measured gene expression after Dox treatment of chondrocytes by qRT‐PCR. The genes expression associated with senescence (*Cdkn1a, Cdkn2a*, and *Tp53*), inflammation (*Il6*, a typical SASP), and cartilage ECM degradation (*Mmp3, Mmp13, Adamts4*, and *Adamts5*) was elevated in the Dox group compared to the control group, whereas the genes expression related to the chondrogenic phenotype (*Col2a1, Aggrecan*, and *Sox9*) was decreased (Figure S16, Supporting Information). We also examined protein expression in chondrocytes after Dox treatment. WB showed that the expression of aggrecan, COL2A1, and SOX9 was decreased in the Dox group, while the expression of p53, p21, and p16^INK4a^ was increased (Figure S17, Supporting Information). Immunofluorescence (IF) staining of p21 and p16^INK4a^ acquired similar results with WB (Figure S18, Supporting Information). Thus, the model of DOX‐induced chondrocyte senescence was used for the experiments in vitro.

### 2.3 Remodeling Efferocytosis via A‐Lipo‐Induced Sn‐Chondrocyte Apoptosis

Senescence upregulates the expression of genes encoding proteins associated with anti‐apoptotic signaling networks, such as the BCL family.^[^ ^36^ ^]^ Consequently, Sn‐chondrocytes undergoing senescence rather than apoptosis are unable to be eliminated by efferocytosis. ABT263, a dual inhibitor of BCL‐2 and BCL‐XL, promotes apoptosis of Sn‐chondrocytes.^[^ ^6b^ ^]^ However, ABT263's poor water solubility limits its application. Here, we prepared A‐Lipo to confirm its ability to induce apoptosis of Sn‐chondrocytes in vitro.

**Figure 3:**
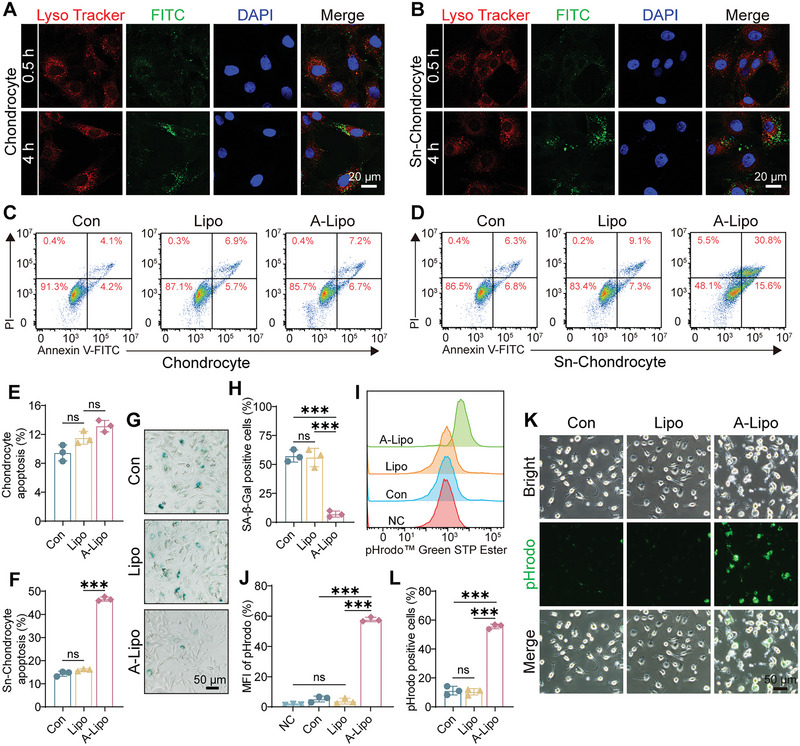
A‐Lipo promoted Sn‐chondrocytes apoptosis and restored the efferocytosis of BMDMs in vitro. A,B) Lysosomal escape assay of A‐Lipo against chondrocytes and Sn‐chondrocytes. Scale bar: 20 µm. C,D) Representative flow cytograms to measure apoptosis of chondrocytes or Sn‐chondrocytes with different treatments. E,F) Quantification of apoptotic cells in figure C&D (*n* = 3). G) Representative images of SA‐β‐Gal staining of Sn‐chondrocytes. Scale bar: 50 µm. H) Quantification of SA‐β‐Gal positive cells. I,J) Representative flow cytograms of the efferocytosis of BMDMs in vitro and quantitative analysis of Mean fluorescence intensity (MFI). NC (Negative Control group; without pHrodo^TM^ Green STP Ester treatment) (*n* = 3). K) Representative microscopic images of BMDMs efferocytosis in vitro with different treatments. BMDMs (Bright), pHrodo^TM^ Green STP Ester (Green). Scale bar: 50 µm. L) Quantification of pHrodo^TM^ Green STP Ester positive cells (*n* = 3). Data are presented as mean ± SD. One‐way ANOVA and Tukey's multiple‐comparisons test were used for data analysis. ns: no significance, ***P < 0.001.

Liposomes are taken up by cells through lysosome‐mediated endocytosis.^[^ ^37^ ^]^ Lysosomal escape assays were employed to assess A‐Lipo uptake by chondrocytes and Sn‐chondrocytes. After 30 min of uptake, we observed a partial overlap between green fluorescence (FITC‐labeled liposomes) and red fluorescence (labeled lysosomes) in both chondrocytes and Sn‐chondrocytes by laser confocal microscopy. After 4 h, green fluorescence was distributed throughout the cytoplasm (**Figure** **3**A,B). This result suggested that lysosomal escape of liposomes occurred mainly after the first 30 min. Chondrocyte viability was determined after 1 and 4 days of ABT263 treatment by the CCK‐8 assay. At a concentration of 20 µg mL^−1^, ABT263 showed no apparent toxicity to chondrocytes. However, chondrocyte viability was significantly decreased on day 1 and day 4 with the increased ABT263 concentration of 40 µg mL^−1^ (Figure S19, Supporting Information). According to the loading efficiency and the safe concentration of ABT263 (20 µg mL^−1^) in chondrocytes, the equivalent concentrations of Lipo and A‐Lipo were calculated to be 207 and 227 µg mL^−1^, respectively. Next, live/dead cell staining indicated that ABT263 (20 µg mL^−1^), Lipo (207 µg mL^−1^) and A‐Lipo (227 µg mL^−1^) exhibited good biocompatibility (Figure S20, Supporting Information). Lipo (207 µg mL^−1^) and A‐Lipo (227 µg mL^−1^) were used in vitro experiments. The apoptosis of chondrocytes and Sn‐chondrocytes was analyzed after Lipo and A‐Lipo treatments for 7 days by flow cytometry (Figure 3C–F). Lipo and A‐Lipo did not promote apoptosis of chondrocytes compared with the control group, but A‐Lipo obviously induced apoptosis of Sn‐chondrocytes. The SA‐β‐Gal staining results in Figure 3G,H showed that A‐Lipo significantly reduced the number of Sn‐chondrocytes.

Efferocytosis of bone marrow‐derived macrophages (BMDMs) was then examined after A‐Lipo promoted apoptosis of Sn‐chondrocytes in vitro. pHrodo is an acidic fluorescent dye that is almost non‐fluorescent at a neutral pH. However, it becomes increasingly fluorescent as the pH decreases, making it suitable for tracking cellular phagocytic processes.^[^ ^38^ ^]^ To study the phagocytosis of apoptotic Sn‐chondrocytes, Lipo or A‐Lipo was applied to Sn‐chondrocytes that had taken up pHrodo Green STP Ester, followed by co‐culture with BMDMs. Flow cytometry showed that the fluorescence intensity of the A‐Lipo group was significantly increased compared with that of the other groups (Figure 3I,J). Fluorescence microscopy images in Figure 3K revealed that the obvious green fluorescence of the A‐Lipo group overlapped with the BMDMs observed by light microscopy. pHrodo‐positive cells were significantly more prevalent in the A‐Lipo group than in the other groups (Figure 3L). These results demonstrated that Sn‐chondrocytes were phagocytosed by BMDMs after treatment with A‐Lipo.

**Figure 4:**
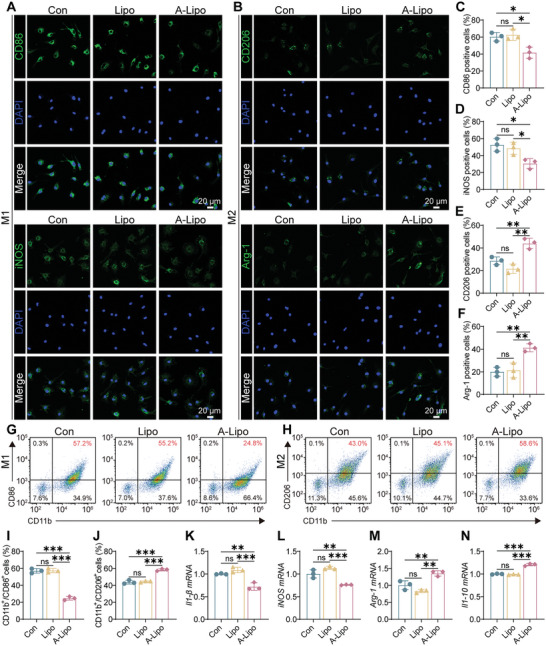
Efferocytosis facilitated BMDMs M2 polarization in vitro. A,B) IF images of BMDMs. Green (M1 marker: CD86 and iNOS; M2 marker: CD206 and Arg‐1), blue (DAPI). Scale bar: 20 µm. C–F) Percentage of positive cells of IF (CD86, iNOS, CD206, and Arg‐1) (*n* = 3). G,H) Representative flow cytograms of CD86 (M1) and CD206 (M2) with different treatments. I,J) Quantitative analysis of positive cells of the flow cytograms (*n* = 3). K–N) Quantitative analysis of mRNA expression of *Il‐1β*, *iNOS*, *Arg‐1*, and *Il‐10* of BMDMs in different groups (*n* = 3). Data are presented as mean ± SD. One‐way ANOVA and Tukey's multiple‐comparisons test were used for data analysis. ns: no significance, *P < 0.05, **P < 0.01, ***P < 0.001.

Efferocytosis consists of three main phases: smell, engulf, and digest phases.^[^ ^2^ ^]^ This process promotes transition of naïve macrophages to the M2 phenotype,^[^ ^2^, ^39^ ^]^ which is accompanied by the release of anti‐inflammatory cytokines. Polarization of BMDMs was assessed after phagocytosis of apoptotic Sn‐chondrocytes using IF, flow cytometry, and qRT‐PCR. As depicted in **Figure** **4**A–F, the positive cell number for M1 markers (CD86 and iNOS) was decreased in the A‐Lipo group, whereas the positive cell number for M2 markers (CD206 and Arg‐1) was increased. Flow cytometry confirmed that CD86‐positive cell count was decreased, and CD206‐positive cell count was increased after treatment with A‐Lipo (Figure 4G–J). We also confirmed that M1‐related genes (*Il‐1β* and *iNOS*) expression was decreased (Figure 4K,L) and M2‐related genes (*Arg‐1* and *Il‐10*) expression was increased (Figure 4M,N) after phagocytosis of apoptotic Sn‐chondrocytes by BMDMs.

In the field of drug delivery, precision targeting strategies are pivotal for achieving specific cellular uptake, thereby minimizing off‐target effects and toxicities.^[^ ^40^ ^]^ Unfortunately, the present study encountered challenges in formulating A‐Lipo with Sn‐chondrocyte targeting capability, largely attributed to the scarcity of reported senescent chondrocyte‐specific targeting molecules. Future research will focus on the targeted elimination of senescent chondrocytes. Macrophages exhibit high‐capacity efferocytosis, with distinct phagocytic capacities across different phenotypes.^[^ ^1b^ ^]^ Compared to the naive macrophages, the phagocytic capacity of M1 macrophages decreased. M2 macrophages show increased phagocytic capacity.^[^ ^2^, ^41^ ^]^ It is worth noting that aging‐related osteoarthritis may lead to a reduction in macrophage phagocytosis to some extent.^[^ ^41a,b^ ^]^ This work mainly aimed to remodel efferocytosis of macrophage after induction of Sn‐chondrocyte apoptosis. Indeed, it is of great significance to investigate how to effectively enhance macrophage phagocytosis in the senescent microenvironment in the future. Senescent cells, which are often referred to as “zombie” cells, cannot be eliminated by the immune system.^[^ ^42^ ^]^ This study emphasizes the significance of promoting apoptosis of Sn‐chondrocytes and their subsequent removal by efferocytosis, which has the potential for treatment of aging‐related diseases.

### 2.4 Inhibition of Chondrocyte Damage by Sn‐Chondrocyte Apoptosis‐Induced Efferocytosis

**Figure 5:**
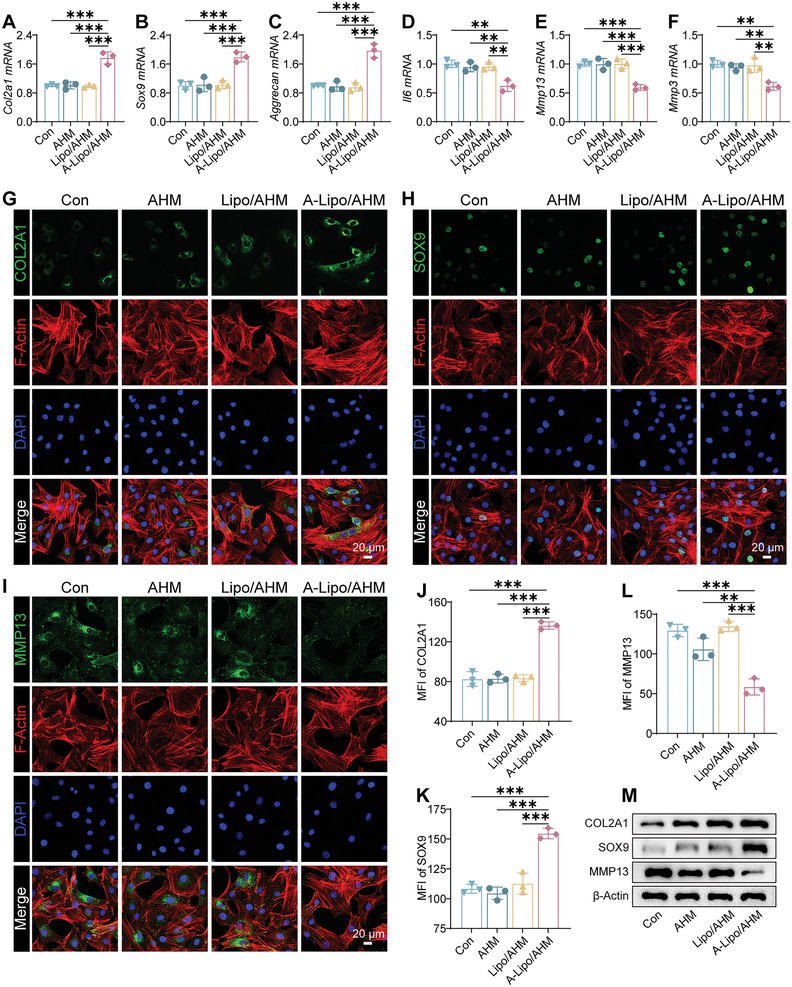
Sn‐chondrocytes apoptosis promoted by A‐Lipo/AHM alleviated chondrocytes damage in vitro. A–F) The mRNA expression of *Col2a1, Sox9, Aggrecan, Il‐6, Mmp13, and Mmp3* of normal chondrocytes in different groups (*n* = 3). G–I) Representative IF images. Green (COL2A1, SOX9, MMP13), Red (F‐Actin), Blue (DAPI). Scale bar: 20 µm. J–L) Quantification of MFI (COL2A1, SOX9, MMP13) (*n* = 3). M) Western blotting analysis of COL2A1, SOX9, and MMP13. Data are presented as mean ± SD. One‐way ANOVA and Tukey's multiple‐comparisons test were used for data analysis. **P < 0.01, ***P < 0.001.

SASP of Sn‐chondrocytes impairs the function of normal chondrocytes. Xu et al. induced OA in mice by injecting senescent auricular chondrocytes into the articular cavity.^[^ ^43^ ^]^ A‐Lipo released from A‐Lipo/AHM promoted Sn‐chondrocytes apoptosis. We assessed the toxicity of AHM or A‐Lipo/AHM with normal chondrocytes. Live/dead cell staining demonstrated good biocompatibility of AHM and A‐Lipo/AHM (Figure S20, Supporting Information). To assess the effect on normal chondrocytes after the removal of Sn‐chondrocytes by efferocytosis, we treated Sn‐chondrocytes with AHM, Lipo/AHM, or A‐Lipo/AHM and cultured normal chondrocytes in a medium consisting of medium culturing Sn‐chondrocytes with different treatments and fresh complete medium. According to the qRT‐PCR results, the expression of chondrocyte‐specific genes (*Col2a1*, *aggrecan* and *Sox9*) was upregulated in the A‐Lipo/AHM group compared with the other groups, while the gene expression related to cartilage ECM degradation (*Mmp3* and *Mmp13*) and an inflammation‐related gene (*Il6*) was decreased compared with the other groups (**Figure** **5**A–F). Cartilage‐specific proteins (COL2A1 and SOX9) and MMP13 protein were further evaluated by IF and WB (Figure 5G–M). IF staining and WB consistently showed that COL2A1 and SOX9 protein expression was higher, and MMP13 protein expression was decreased in the A‐Lipo/AHM group. Therefore, the clearance of Sn‐chondrocytes by A‐Lipo/AHM contributed to the protection of normal chondrocytes.

### 2.5 Assessment of Stem Cell Recruitment and Maintenance of Chondrogenic Differentiation of BMSCs by Sn‐Chondrocyte Apoptosis‐Induced Efferocytosis

**Figure 6:**
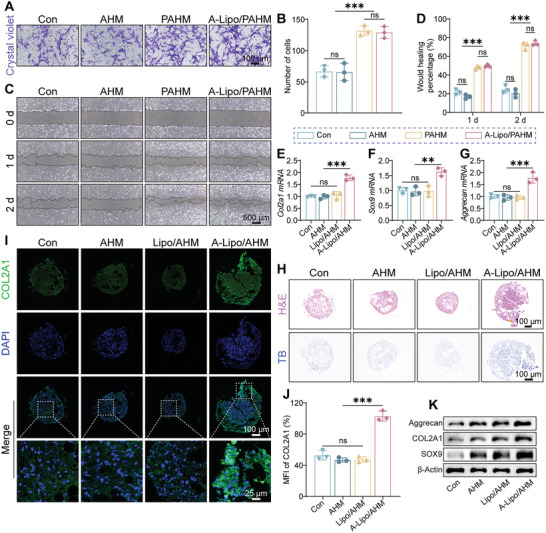
Assessment of stem cell recruitment ability of microspheres in vitro and chondrogenic differentiation capability of BMSCs after Sn‐chondrocytes apoptosis. A) Representative images of crystal violet staining of BMSCs for chemotaxis experiments. Scale bar: 100 µm. B) Quantification of the number of migrating cells (*n* = 3). C) Representative microscopic images of the wound‐healing assay of BMSCs. Scale bar: 500 µm. D) Quantification of the percentage of wound‐healing (*n* = 3). E–G) The mRNA expression of *Col2a1, Sox9*, and *Aggrecan* of BMSCs pellets with different treatments (*n* = 3). H) Images of H&E staining and Toluidine blue (TB) staining of BMSCs pellets in different groups. I) Representative images of COL2A1 IF staining of BMSCs pellets. J) Quantification of MFI (COL2A1) (*n* = 3). K) Western blotting analysis of Aggrecan, COL2A1, and SOX9 of BMSCs pellets. Data are presented as mean ± SD. One‐way ANOVA and Tukey's multiple‐comparisons test were used for data analysis. ns: no significance, **P < 0.01, ***P < 0.001.

SASP of Sn‐chondrocytes hinders the chondrogenic differentiation capacity of stem cells. Yang et al. found that clearance of joint senescent cells resulted in better cartilage regeneration by stem cells in rats.^[^ ^12b^ ^]^ Before assessment of the chondrogenic differentiation ability, we indirectly assessed the stem cell recruitment ability of microspheres containing PDGF‐BB in vitro by cell chemotaxis and scratch assays. In the chemotaxis assay, AHM, PAHM, and A‐Lipo/PAHM were placed in the lower chamber of transwells, while BMSCs were seeded in the upper chamber. After 48 h, more cells stained blue by crystal violet were observed by light microscopy in PAHM and A‐Lipo/PAHM groups (**Figure** **6**A,B). Furthermore, as depicted in Figure 6C,D, the cell migration area was increased in all groups at 1 and 2 days, with the most notable healing rate observed in PAHM and A‐Lipo/PAHM groups, which showed healing rates of 71.3% and 74.0%, respectively, at two days. Next, we treated stem cell pellets with a medium consisting of medium culturing Sn‐chondrocytes with different treatments and chondrogenic induction medium for 14 days, followed by evaluation of BMSC chondrogenic differentiation. qRT‐PCR results in Figure 6E–G showed that gene expression of *Col2a1*, *aggrecan* and *Sox9* was higher in the A‐Lipo/AHM group than that in the other groups. After being fixed, embedded, and sectioned, the structure of BMSC pellets differentiated into chondrocytes was observed by HE staining (Figure 6H). Toluidine blue (TB) staining and IF staining of COL2A1 showed significantly higher expression of glycosaminoglycans and COL2A1 in the A‐Lipo/AHM group (Figure 6H–J). WB showed that aggrecan, COL2A1 and SOX9 protein expression was higher in the A‐Lipo/AHM group than in the other groups (Figure 6K). Therefore, the microspheres prepared in this study recruited stem cells and maintained the chondrogenic differentiation ability of stem cells by eliminating Sn‐chondrocytes.

### 2.6 Evaluation of the Efficacy of Lesion‐Localized Hydrogel Microspheres for the Treatment of OA In Vivo

To evaluate the retention time of agents in the joint, we injected DiD‐Lipo (DiD‐labeled liposomes) and DiD‐Lipo/AHM into the mouse knee joint, observed the fluorescence intensity of the bilateral knee joints, and quantified the relative fluorescence intensity using an IVIS‐spectrum system. The fluorescence intensity of the left knee joint of mice injected with DiD‐Lipo decreased rapidly at one week, and almost no fluorescence was observed at 14 days. However, the right knee joint of mice injected with DiD‐Lipo/AHM showed weak fluorescence at 28 days, suggesting that the loading of liposomes into microspheres prevented rapid clearance (Figure S21, Supporting Information). The therapeutic efficacy of agents is closely related to their retention time in the joint.^[^ ^17b^ ^]^ The ability of the synovial membrane and lymphatic system to remove drugs is closely related to the particle size.^[^ ^17b^ ^]^ The half‐life of NSAIDs and glucocorticoids is 1–4 h, and hyaluronic acid is completely cleared within 26 h.^[^ ^44^ ^]^ Nanoparticles do not remain in the joint for long periods.^[^ ^45^ ^]^ The hydrogel microspheres were designed to adhere to damaged cartilage and slowly release liposomes for a prolonged anti‐senescence effect.

**Figure 7:**
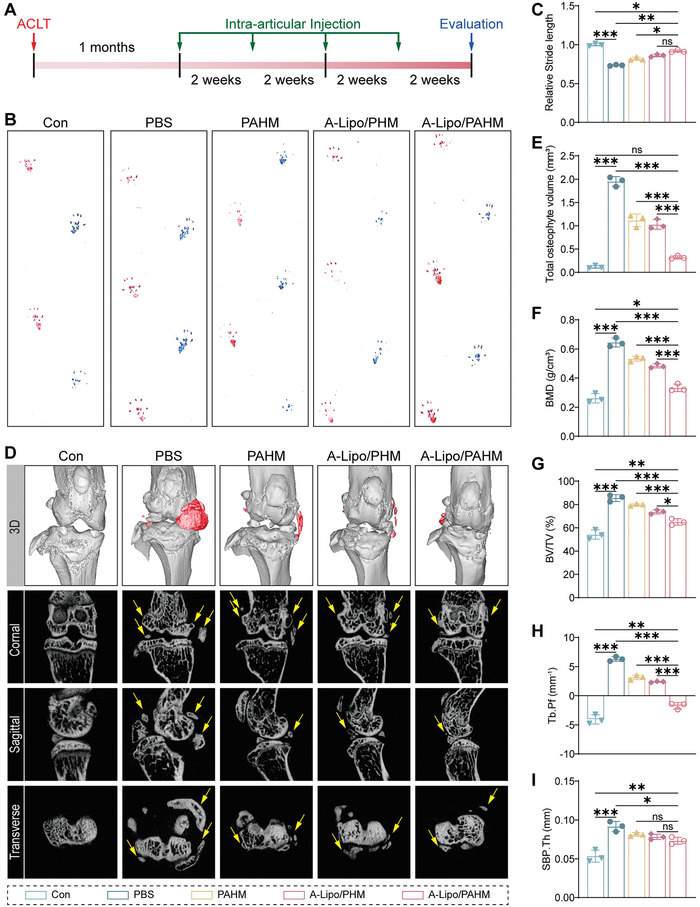
Gait analysis and microCT to evaluate the OA treatment effect in vivo. A) Scheme of the in vivo experiment with a mouse model. B) Footprints of mice after 2 months of treatment, normal foot (red), experimental foot (blue). C) Relative stride length of mice in different groups (*n* = 3). D) 3D reconstruction images, coronal, sagittal, and transverse images of mouse knee microCT. E–I) Osteometry parameters of knee microCT (*n* = 3). total osteophyte volume (mm^3^), BMD (g/cm^3^), BV/TV %, Tb.Pf (mm^−1^), SBP.Th (mm). Data are presented as mean ± SD. One‐way ANOVA and Tukey ’s multiple‐comparisons test were used for data analysis. ns: no significance, *P < 0.05, **P < 0.01, ***P < 0.001.

Next, an anterior cruciate ligament transection (ACLT)‐induced mouse OA model was used in vivo.^[^ ^12a^ ^]^ One month after surgery, knee joints were injected with PBS, PAHM, A‐Lipo/PHM, and A‐Lipo/PAHM every two weeks, and therapeutic efficacy was assessed at three months after surgery (**Figure** **7**A). To assess the recruitment of endogenous stem cells, some of these knee samples were collected to perform immunofluorescence staining of CD90, which is a marker of MSCs. Compared to the Control group and PBS group, CD90 positive cells were significant observed in the treatment groups (Figure S22A, Supporting Information). The percentage of CD90 positive cells was also quantified and the results indicated that microspheres loaded with PDGF‐BB were able to attract endogenous stem cells to the degraded cartilage surface in OA (Figure S22B, Supporting Information). Images of mouse footprints were obtained. Red footprints represented the healthy side, and blue footprints represented the model side (Figure 7B). We performed a quantitative analysis of the relative stride length with these footprint images. Although relative stride length was not significant between the A‐Lipo/PAHM and A‐Lipo/PHM groups, the relative stride length was increased in the A‐Lipo/PAHM group compared with the PBS group (Figure 7C). The stride length of the lower limbs decreased due to cartilage damage and joint pain in OA, and was restored with OA relief. X‐ray imaging and microCT were also used to assess OA severity. On plain radiographs and 3D or 2D images of microCT, we observed obvious osteophyte formation in the PBS group compared with the control group. After treatment, the reduction of osteophytes was most evident in the A‐Lipo/PAHM group compared with the other treatment groups (Figure 7D,E; Figure S23, Supporting Information). Subsequently, the osteometric parameters of the subchondral bone were analyzed by microCT (Figure 7F–I). BMD, BV/TV, Tb.Pf and SBP.Th was decreased in the experimental group compared to the PBS group, with the most significant decrease in the A‐Lipo/PAHM group.

To assess the microsphere biocompatibility in vivo, after sacrificing the animals, the heart, liver, spleen, lung, and kidney tissues were obtained and stained with H&E. No significant histological alterations were found in the experimental groups compared to the control group, indicating that local administration of the microspheres into the joint was safe (Figure S24, Supporting Information).

**Figure 8:**
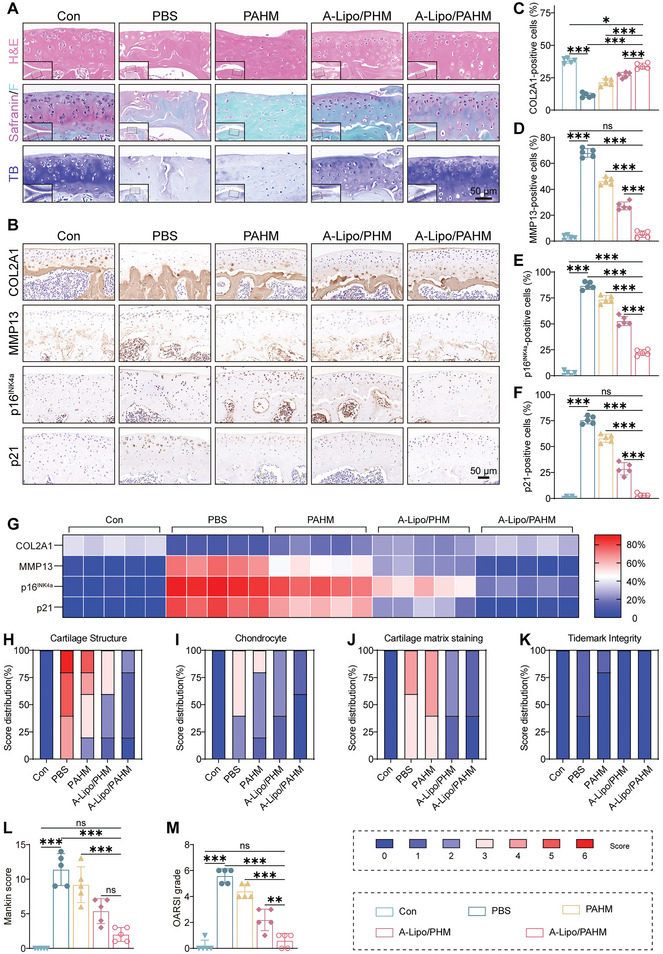
Histological staining and histological scoring to evaluate the OA treatment effect in vivo. A) Images of H&E staining, Safranin‐O/fast green staining, and Toluidine blue (TB) staining. Scale bar: 50 µm. B) Representative images of immunohistochemical (IHC) staining of COL2A1, MMP13, p16^INK4a^, and p21. Scale bar: 50 µm. C–F) Percentage of IHC positive cells (COL2A1, MMP13, p16^INK4a^, p21) (*n* = 5) G) Heatmap of the percentage of COL2A1, MMP13, p16^INK4a^, and p21 positive cells (*n* = 5). H–K) Detailed score distribution of Mankin score in term of cartilage structure, chondrocytes, cartilage matrix staining, and tidemark integrity. L) Mankin score of the mice knee joints based on tissue staining images (*n* = 5). M) OARSI grades of the mice knee joints (*n* = 5). Data are presented as mean ± SD. One‐way ANOVA and Tukey's multiple‐comparisons test were used for data analysis. ns: no significance, *P < 0.05, **P < 0.01, ***P < 0.001.

Histological staining and scoring were used to further evaluate the therapeutic effect in vivo (**Figure** **8**). As shown in Figure 8A, H&E staining demonstrated that chondrocytes were well arranged, the cartilage surface was flat, and the thickness of the cartilage matrix was uniform in the control group. However, in the PBS group, the chondrocytes were significantly reduced, the surface of the cartilage was rough, and the subchondral bone was exposed. Among the treatment groups, chondrocytes and cartilage matrix were maintained in the A‐Lipo/PAHM group. Toluidine blue and Safranin‐O/fast green staining showed that A‐Lipo/PAHM significantly alleviated cartilage destruction (Figure 8A). Immunohistochemical staining was then used to assess cartilage synthesis, catabolism, and senescence. We observed a significant increase in COL2A1 expression and a decrease in MMP13, p16^INK4a^, and p21 expression in the A‐Lipo/PAHM group compared with the other experimental groups (Figure 8B). The percentage of positive cells in immunohistochemistry images was determined (Figure 8C–F). Compared with the PBS group, COL2A1‐positive cells were significantly increased, and MMP13‐, p16^INK4a^‐ and p21‐positive cells were significantly decreased in the A‐Lipo/PAHM group. Besides, the immunohistochemical results were presented in a heatmap (Figure 8G). Using the semi‐quantitative scoring system recommended by OARSI,^[^ ^46^ ^]^ the score of the PBS group was significantly increased compared with that of the control group, and the score decreased after treatment with the most significant decrease in the A‐Lipo/PAHM group (Figure 8M). The results of the Mankin score calculated by comparing the cartilage structure, chondrocytes, matrix staining, and tideline integrity of each group were consistent with those of the OARSI score (Figure 8H–L).^[^ ^47^ ^]^

Compared with oral medications, intra‐articular injection of agents for OA treatment increases drug utilization and avoids the risk of systemic adverse effects of the drug.^[^ ^48^ ^]^ However, the drug residence time in the joint is very short due to clearance by the synovial membrane. To improve the therapeutic effect, repeated intra‐articular injections increase the risk of joint infection and even lead to joint disability.^[^ ^45^ ^]^ Therefore, efficient drug delivery systems are needed to overcome these problems. Hydrogel microspheres are a novel drug delivery system because of their biocompatibility, injectability, and ease of modification, which allows for prolonged drug release for OA treatment.^[^ ^30^, ^49^ ^]^

## 3 Conclusion

In summary, we designed a hydrogel microsphere localized to lesioned cartilage to reverse cartilage aging for OA treatment via remodeling efferocytosis. The prepared aldehyde‐modified microspheres were proven to adhere to degraded cartilage with an OA model of porcine cartilage explant, enabling a precise cartilage location strategy in OA. In vitro, A‐Lipo promoted apoptosis of Sn‐chondrocytes, which induced the efferocytosis of BMDMs to eliminate Sn‐chondrocytes. With the clearance of Sn‐ chondrocytes, the characteristics of normal chondrocytes and the chondrogenic differentiation capacity of stem cells were maintained. The in vivo experiments confirmed the recruitment of stem cells and the effect of reversing cartilage senescence and promoting cartilage repair in an ACLT‐induced OA model. Therefore, the reversal of senescence by remodeling efferocytosis may be a general treatment strategy for aging‐related diseases and provided a broad perspective in the field of aging.

## 4 Experimental Section

All animal husbandry and experiments were approved by the Animal Ethics Committee of Shanghai Shengchang Biotechnology Co., Ltd (2023‐02‐SGKYJS‐CWG‐042). and strictly followed the National Institutes of Health Guidelines for the Care and Use of Laboratory Animals. The experimental details of the synthesis and characterization of the materials, in vitro and in vivo experiments are available in the Supporting Information.

## Conflict of Interest

The authors declare no conflict of interest.

## Supporting information

Supporting Information

## Data Availability

The data that support the findings of this study are available from the corresponding author upon reasonable request.
