# Supplementary material for: In Situ Remodeling of Efferocytosis via Lesion‐Localized Microspheres to Reverse Cartilage Senescence
Source: Adv Sci (Weinh). 2024 Mar 13;11(19):2400345. doi: 10.1002/advs.202400345 (PMC11109622; doi:10.1002/advs.202400345)
Supplement: Supplementary file 1 — Supporting Information [file ADVS-11-2400345-s001.pdf]

ADVANCED SCIENCE
Open Access

Supporting Information

for *Adv. Sci.*, DOI 10.1002/advs.202400345

In Situ Remodeling of Efferocytosis via Lesion-Localized Microspheres to Reverse Cartilage Senescence

Wei Xiong, Zeyu Han, Sheng-Long Ding, Haoran Wang, Yawei Du, Wenguo Cui* and Ming-Zhu Zhang**

Supporting Information

In situ remodeling of efferocytosis *via* lesion-localized microspheres to reverse cartilage senescence

Wei Xiong, Zeyu Han, Sheng-Long Ding, Haoran Wang, Yawei Du, Wenguo Cui*, Ming-Zhu Zhang**

Dr. W. Xiong, Dr. Z. Han, Dr. S. Ding, Prof. M. Zhang
Department of Foot and Ankle Surgery, Beijing Tongren Hospital, Capital Medical University, Beijing 100730, P. R. China
E-mail: michaelzhang120@hotmail.com

Dr. W. Xiong, Dr. Z. Han, Dr. H. Wang, Dr. Y. Du, Prof. W. Cui
Department of Orthopaedics, Shanghai Key Laboratory for Prevention and Treatment of Bone and Joint Diseases, Shanghai Institute of Traumatology and Orthopaedics, Ruijin Hospital, Shanghai Jiao Tong University School of Medicine, 197 Ruijin 2nd Road, Shanghai 200025, P. R. China.
E-mail: ywdu@hotmail.com; wgcui80@hotmail.com

Keywords: Hydrogel microspheres, lesion-localized, efferocytosis, cartilage senescence, osteoarthritis

Contents

Experimental section

Preparation and characterization of liposomes

Synthesis and characterization of HAMA and AHAMA hydrogels

Synthesis and characterization of A-Lipo/PHM and A-Lipo/PAHM

Cell isolation and culture

Induction of chondrocyte senescence *in vitro*

CCK-8 assays

Live/dead cell staining

Lysosomal escape assay

SA-β-Gal staining

Efferocytosis assay

Flow cytometry

Quantitative reverse transcription-polymerase chain reaction (qRT-PCR)

Immunofluorescence (IF)

Western blotting (WB)

BMSCs migration assay

Wound-healing assay

BMSCs chondrogenic differentiation assay

Establishment of the OA mouse model

In vivo fluorescence imaging

Gait analysis

X-ray and microCT

Histological assessment

Immunohistochemical staining

Statistical analysis

References

Supplementary Figures

Figure S1. Stability of Lipo in vitro over 28 days.

Figure S2. Stability of A-Lipo *in vitro* over 28 days.

Figure S3. The cumulative release profile of ABT263

Figure S4. Schematic diagram of the chemical reaction between AHA and MA

Figure S5. Schematic diagram of the chemical reaction between HA and MA

Figure S6. (A) HM images acquired with a bright-field microscope.

Figure S7. Representative SEM images of HM.

Figure S8. *In vitro* degradation properties of AHM and HM

Figure S9. The Zeta potentials of HM, AHM, A-Lipo/HM, A-Lipo/AHM, A-Lipo/PHM, and A-Lipo/PAHM.

Figure S10. The PDGF-BB loading efficiency of AHM and A-Lipo/AHM.

Figure S11. The PDGF-BB loading efficiency of HM and A-Lipo/HM.

Figure S12. Measuring the cumulative release rate of Lipo within Lipo/PHM under PBS or PBS containing HAase

Figure S13. Measuring the cumulative release rate of PDGF-BB within Lipo/PHM under PBS or PBS containing HAase

Figure S14. Representative fluorescence images of BMSCs cultured on HM, AHM, A-Lipo/AHM, and A-Lipo/PAHM.

Figure S15. Images of SA-β-Gal staining of chondrocytes.

Figure S16. Quantitative analysis and heatmap of mRNA expression after 14 days of Dox intervention.

Figure S17. Western blotting analysis after 14 days of Dox intervention.

Figure S18. Representative immunofluorescence images of $P16^{INK4a}$ and P21

Figure S19. Cell viability analysis of chondrocytes after treatment with ABT263

Figure S20. Live/dead cell staining of chondrocytes

Figure S21. IVIS images at different time points *in vivo*.

Figure S22. Assessment of the ability of microspheres to recruit stem cells *in vivo*.

Figure S23. Representative X-ray films of the mice knee joints

Figure S24. To assess the biocompatibility of microspheres *in vivo*.

Supplementary Table

Table S1. List of primers for qRT-PCR

Experimental section

Preparation and characterization of liposomes

The thin film dispersion method was utilized for liposome preparation. Briefly, cholesterol (Aladdin, China), lecithin (Macklin, China), and ABT263 (Aladdin, China) were dissolved in dichloromethane at a mass ratio of 8:2:1. After vacuum rotary evaporation at 38 °C for half an hour, the dried lipid membranes were hydrated using PBS and sonicated for 10 minutes. Liposomes were obtained after repeated extrusion using polycarbonate membranes (0.45 and 0.22 μm, Millex, Ireland). To prepare fluorescently labeled liposomes, DSPE-PEG-FITC (Xi'an Ruixi Biology, China) or DiD (Beyotime, China) was added during the synthesis process.

To observe the liposome morphology, Lipo or A-Lipo (0.5 mg/mL) was dripped onto a TEM copper mesh (300 mesh) and allowed to dry completely. It was then negatively stained with 1% phosphotungstic acid (Solarbio, China) for 1 min and allowed to dry again for TEM (Hitachi-HT7800, Japan) observation. The size distribution, PDI, and zeta potential of Lipo and A-Lipo were determined by DLS (Zetasizer Nano-ZS, Malvern, UK). The stability of Lipo and A-Lipo in a physiological environment was assessed by dissolving them in 50% FBS (BioInd, Israel) at 37 °C and their size and PDI was measured by DLS at 1, 3, 5, 7, 14, 21, and 28 days. To calculate drug encapsulation (EE) and loading efficiency (LE), the liposome structure was disrupted using methanol and DMSO at a volume ratio of 1:9, and then the amount of ABT263 in A-Lipo was calculated using a concentration-OD value standard curve of ABT263. The formula to calculate the EE and LE of ABT-263 was as follows:

$$\mathbf{EE\ (\%)=\frac{W}{W_0}\times 100\%}$$

(W: mass of ABT263 loaded on the liposomes; W_0: total mass of drug added to the reaction)

$$\mathbf{LE\ (\%)=\frac{W}{W+W_{Lipo}}\times 100\%}$$

(W_{Lipo} mass of liposomes)

Release behavior of ABT263 *in vitro*. A-Lipo was put in a dialysis bag (MW =

3500 Da) and dialyzed against PBS containing 1% Tween 80. At the appropriate time points, 1 ml of the dialyzed sample was gathered, and an equivalent amount of fresh PBS with 1% Tween 80 was added. The OD values of the solutions collected at each time point were measured using a UV spectrophotometer (Jasco, Japan). The cumulative release rate was calculated, and a release curve was plotted.

Synthesis and characterization of HAMA and AHAMA hydrogels

AHA was synthesized using the previously reported method.[1] One gram of HA (MW = 74 kDa) was dissolved in 100 ml ultrapure water with mechanical stirring until complete dissolution. Then, 5 ml of 0.5 M $NaIO_3$ (Macklin, China) was pipetted dropwise, and the reaction was carried out for two hours. Subsequently, ethylene glycol (Macklin, China) was added to terminate the reaction. AHA was obtained by lyophilization after three days of dialysis.

HAMA and AHAMA hydrogels were synthesized as described previously.[2] Briefly, 10 g HA or AHA was dissolved in 500 ml ultrapure water to be completely transparent. Then, 20 ml MA (Sigma-Aldrich, USA) was added dropwise and mixed well. A NaOH solution (20 mL, 5 M) was added using a micro-syringe pump. The reaction was conducted at 4 °C while protected from light overnight. After the reaction, HAMA or AHAMA was obtained by lyophilization after three days of dialysis.

HA, AHA, HAMA, and AHAMA were characterized using a Fourier-transform infrared spectrometer (FTIR, Nicolet is50, Thermo Fisher, USA) and ^{1}H NMR (600 MHz, Bruker, Germany). [3] The grafting rate of MA is calculated by ^{1}H NMR. The MA grafting rate was calculated from the integral areas of proton peaks attributed to ethylenic bond groups of grafting MA side chain (S_1, chemical shift 5.7 and 6.1 ppm) and methyl groups of MA side chain and HA or AHA backbone (S_2, chemical shift 1.8 and 1.9 ppm) using the formula as follows:

$$\textbf{The grafting rate of MA (\%)} = \frac{\frac{S_1}{2}\times 3}{S_2-\frac{S_1}{2}\times 3}\times 100\%$$

(S_1: integral area of ethylenic bond proton peaks; S_2: integral area of methyl proton peaks)

The content of aldehyde groups in AHA and AHAMA was determined by hydroxylamine hydrochloride titration [4] and XPS. 0.1 g of the sample was dissolved in ultrapure water until complete dissolution, and the pH value of the solution was adjusted to 5.0 with NaOH solution (0.1 M), then hydroxylamine hydrochloride solution (0.05 g/mL) was added for 4 h reaction at 40°C. At last, the pH value of the reaction solution was adjusted to 5.0 again with NaOH solution, and the volume of NaOH solution used in this step was recorded. The calculation formula is as follows:

$$\textbf{The oxidation level (\%)}=\frac{\mathbf{M\times(V\times n_{NaOH})}}{\mathbf{2m}}\times\mathbf{100\%}$$

(M (g/mol): the monomer unit molar mass of HA or HA grafted MA, V (L): the volume of NaOH solution recorded, n_{NaOH} (mol/L): the concentration of the NaOH solution, m (g): mass of sample to be measured)

The monomer unit molar mass of HA is 376 g/mol, and the average monomer unit molar mass calculated from the grafting rate of MA was used as the monomer unit molar mass of HA grafted MA.

Synthesis and characterization of A-Lipo/PHM and A-Lipo/PAHM

Hydrogel microspheres were prepared by microfluidic technology and a photo-crosslinking method.[5] Briefly, AHAMA or HAMA, A-Lipo, and LAP (Sigma-Aldrich, USA) (4%/0.2%/0.2%, mass ratio) were used as the dispersed phase, and the continuous phase used was liquid paraffin with 5% Span 80. Using an in-house microfluidic device, the dispersed phase was sheared into microdroplets by the continuous phase. After being collected at -20 °C and frozen overnight at -80 °C, the microdroplets were photo-crosslinked by a 405 nm UV light for 10 min. Microspheres were collected, washed with diethyl ether and ultrapure water, and then lyophilized for use in subsequent experiments. To load PDGF-BB, 1 mg dried microspheres were co-incubated with PDGF-BB (300 ng/mL; Novoprotein, China) in a shaker at 4 °C overnight. Precipitates were collected to obtain A-Lipo/PHM or A-Lipo/PAHM.

The size and morphology of microspheres were observed by optical microscopy and SEM (Hitachi Regulus8100, Japan). The size of microspheres was measured using

ImageJ. The zeta potential of microspheres was measured by DLS. Microspheres loaded with FITC-labeled liposomes were observed by laser confocal microscopy (LSM800, ZEISS, Germany). To assess the swelling properties of microspheres, 10 mg of dry microspheres were added to 2 mL of PBS, and the mass of the microspheres was weighed after removing as much residual water as possible at various time points. The swelling ratio of microspheres was calculated as follows:

$$\textbf{Swelling ratio (\%)}=\frac{\mathbf{W_{wet}}}{\mathbf{W_{dry}}}\times\mathbf{100\%}$$

(W_{wet}: mass of wet microspheres after water absorption, W_{dry}: mass of dry microspheres)

Evaluation of the degradation properties of microspheres. The lyophilized microspheres were resuspended in PBS containing hyaluronidase (1500 U/mL, Macklin, China), and the morphology of the microspheres was observed under an optical microscope at various time points. To assess the release behavior of liposomes in microspheres, LS release experiments were performed using microspheres loading with FITC-labeled liposomes. At the corresponding time points, supernatants were harvested, and the intensity of the fluorescence was quantified. To calculate the concentration of released liposomes, a standard curve of fluorescence intensity-concentration was constructed, and then the cumulative release curve of liposomes was plotted. To investigate the EE of PDGF-BB, an absorbance value-concentration standard curve of PDGF-BB was constructed. The PDGF-BB content in the supernatant was determined by ELISA, and the encapsulation efficiency of PDGF-BB was calculated using the encapsulation efficiency formula. To evaluate PDGF-BB release from microspheres, supernatants were collected at various time points, and the PDGF-BB concentration was determined using an ELISA to plot the cumulative release curve of PDGF-BB.

Evaluation of injured cartilage adhesion properties of microspheres. BMSCs were cultured on AHM. Specifically, 1 mg of AHM and 1×10^6/mL BMSCs were added to a low-adhesion plate and cultured for 3 days. Suspended cells were removed with a 70 μm cell sieve (Sangon Biotech, China). The growth of cells stained with Actin-Tracker Green-488 (Beyotime, China) and DAPI (Beyotime, China) on the microspheres was observed by laser confocal microscopy. An OA cartilage model was established by

harvesting healthy full-depth cylindrical cartilage explants (7 mm diameter) and incubating them in 0.1% collagenase (Sigma-Aldrich, USA) for half an hour at 37 °C. The cartilage explants were placed in a solution of 2 mg/mL microspheres, magnetically stirred for 5 min, and then left to stand for 1 min. The cartilage explants were taken out and rinsed with PBS several times. To observe adhered microspheres, digital photographs of the various groups were acquired.

Cell isolation and culture

Extraction of primary mouse chondrocytes. Knee cartilage tissues from 7-day-old C57BL/6 mice were removed, cut into pieces, and digested with 0.5% trypsin-EDTA (Hycolon, USA) for 30 min, followed by incubation with 2 mg/mL collagenase (Sigma-Aldrich, USA) for 8 hours. Incompletely digested tissue was removed using a 70 μm cell sieve (Sangon Biotech, China). The digested cell suspension was cultured in a high glucose DMEM medium (Hyclone, USA) containing 1% penicillin/streptomycin (Biosharp, China) and 10% FBS (BI, Israel) at 37 °C, with 5% CO_2. At 80% confluency, they were passaged at a 1:3 ratio. Passage 2 cells were used for subsequent experiments.

Mouse BMSCs were isolated using a previously reported method. Briefly, the femur and tibia of 7-day-old C57BL/6 mice were removed, their ends were severed, and α-MEM medium (Hyclone, USA) containing 10% FBS and 1% penicillin/streptomycin was used to rinse the bone marrow cavity. The rinsed-out cells were cultured at 37 °C with 5% CO_2. At 80% confluency, the cells were passaged at a 1:3 ratio. Passage 3 cells were used for subsequent experiments.

To isolate mouse BMDMs,[6] femurs and tibiae of 6–8-week-old C57BL/6 mice were extracted, and a cell suspension was collected from the bone marrow cavity as described above. After centrifugation, the cells were resuspended with α-MEM medium containing 20 ng/mL M-CSF (Proteintech, Wuhan, China), 10% FBS, and 1% penicillin/streptomycin, and cultured at 37 °C with 5% CO_2, for which fresh complete medium was substituted on days 3 and 5. BMDMs were collected on day 7 for subsequent experiments.

Induction of chondrocyte senescence *in vitro*

Passage 2 chondrocytes were used to establish a chondrocyte senescence model. Adherent chondrocytes were induced with Dox (100 ng/mL; Sigma-Aldrich, USA) for 14 days. qRT-PCR, WB, and IF were used to determine the successful establishment of the chondrocyte senescence model.

CCK-8 assays

The seeded chondrocytes were treated with various concentrations of ABT263 for 1 and 4 days, and then CCK-8 solution (Beyotime, China) was added. After incubation for 2 hours at 37 °C in the dark, the absorbance of the solution was measured at 450 nm using a microplate reader (Molecular Devices, FlexStation 3).

Live/dead cell staining

Chondrocyte viability was assessed on days 1 and 4 using a Calcein/PI Assay Kit (Beyotime, China). Briefly, washing cells gently, Calcein/PI working solution (300 μL) was added, with which cells were incubated for half an hour at 37 °C in the dark. The cells were observed by the fluorescence microscope, and chondrocyte viability was analyzed using ImageJ.

Lysosomal escape assay

After treating cells with FITC-labeled liposomes for 0.5 and 4 h, the cells were incubated with Lyso-Tracker Red working solution (Solarbio, China) at 37 °C for 10 minutes and Hoechst 33342 (Beyotime, China) was employed to stain nuclei. Images were randomly obtained under a laser confocal microscope.

SA-β-Gal staining

After fixation and washing, cells were incubated with the Senescence β-Galactosidase Staining Working Solution (Beyotimie, China) overnight at 37°C.

Images were obtained randomly with the bright-field microscope. The positive cell number was counted using ImageJ.

Efferocytosis assay

After adding pHrodo™ Green STP Ester (Thermo Fisher Scientific, USA) to the digested cells, they were incubated for 2 hours at RT in the dark. The supernatant was removed after centrifugation, followed by addition of fresh cold complete medium to wash the cells three times. pHrodo™ Green STP Ester-treated cells were co-incubated with adherent BMDMs in a 5:1 ratio for 2 hours. Phagocytosed apoptotic cells were observed by fluorescence microscopy, or cells were collected to detect fluorescence by flow cytometry. Fluorescence was quantified using ImageJ.

Flow cytometry

Detection of apoptosis. Floating and digested cells were collected and stained using an Annexin V-FITC Apoptosis Detection Kit (Beyotime, China). Determination of the BMDM phenotype. Collected BMDMs were incubated with antibodies against a macrophage marker (CD11b), M1-specific markers (CD86 and iNOS), and M2-specific markers (CD206 and Arg-1) for half an hour. After three washes with PBS, cells were analyzed by a flow cytometer (BD, LSRFortessa X-20, USA). Data were managed using FlowJo V 10.0 software.

These antibodies were employed：anti-CD86 antibody (1:200， Thermo Fisher Scientific), anti-iNOS antibody (1:200， Thermo Fisher Scientific), anti-CD206 antibody (1:200， Thermo Fisher Scientific), anti-Arg-1 antibody (1:200， Thermo Fisher Scientific), anti-CD11b antibody (1:200， Thermo Fisher Scientific).

Quantitative reverse transcription-polymerase chain reaction (qRT-PCR)

Total RNA was extracted and reverse transcribed to synthesize cDNA. Following this, the SYBR Green fluorescent dye method was employed for qRT-PCR. Target gene expression was compared to that of the GAPDH gene for normalization. The $2^{-\Delta\Delta Ct}$

method was employed to calculate the relative expression of the target genes. Three biological replicates were included for each target gene. The primers used are shown in Table S1.

Immunofluorescence (IF)

After fixation and blocking, samples were incubated with a primary antibody at 4 °C overnight and then incubated with goat anti-rabbit IgG H&L (Alexa Fluor 488, Beyotime, China) or goat anti-mouse IgG H&L (Alexa Fluor 488, Beyotime, China) for 1 hour at RT. Finally, the nucleus and cytoskeleton are stained with Actin-Tracker Red-555 (Beyotime, China) or DAPI (Beyotime, China). Each staining step requires at least three washes with PBS. Images of fluorescence were captured by the laser confocal microscope. The MFI in each group was analyzed using ImageJ.

These primary antibodies were employed：anti-p16^{INK4a} antibody (1:50, Santa)，anti-p21 antibody (1:50, Santa)，anti-SOX9 antibody (1:1000, Abcam)，anti-COL2A1 antibody (1:50, Santa)，anti-AGGRECAN antibody (1:100, ABclonal)，anti-CD86 antibody (1:100, Abclonal), anti- iNOS antibody (1:20, Abcam), anti-CD206 antibody (1:200, Abcam), anti- Arg-1 antibody (1:250, Abcam), anti-CD90 antibody (1:50, Affinity)

Western blotting (WB)

With extraction of total protein and measurement its protein concentration, target proteins were separated and then transferred to a PVDF membrane. The PVDF membrane with target proteins was incubated in QuickBlock™ Blocking Buffer (Beyotime, China) for half an hour, which was then incubated with a primary antibody overnight at 4 °C, washed three times with Western Wash Buffer (Beyotime, China) and incubated with goat anti-mouse (1:400, Beyotime, China) or goat anti-rabbit (1:400, Beyotime, China) secondary antibodies for 1.5 hours at room temperature. The chemiluminescence detection system (ChemiDoc™ Touch Imaging System, Bio-Rad) was used to visualize protein bands.

These primary antibodies were employed：anti-p16INK4a antibody (1:200, Santa)，anti-p21 antibody (1:200, Santa), anti-p53 antibody (1:200, Santa), anti-SOX9 antibody (1:1000, Abcam), anti-COL2A1 antibody (1:200, Santa), anti-AGGRECAN antibody (1:500, ABclonal), anti-β-Actin antibody (1:400, Beyotime)

BMSCs migration assay

Using a transwell system (Corning, USA), The lower chamber was added with microspheres, and the upper polycarbonate membrane (8 μm pores) was cultured with BMSCs. After two days of incubation, cells crossed the polycarbonate membrane were fixed and stained by crystal violet (Solarbio, China). With the bright-field microscope, the images of crossed cells were obtained, and ImageJ was chosen to calculate the cell number in each group.

Wound-healing assay

At 90% confluency of BMSCs, cell scratching was performed, and images were obtained immediately by the light microscope. After treatment in the various groups, wound healing was observed at 1 and 2 days, and representative images were obtained. The wound healing rate was measured using ImageJ.

BMSCs chondrogenic differentiation assay

Sn-chondrocytes were treated with PBS, AHM, Lipo/AHM, or A-Lipo/AHM for 7 days and then cultured for 3 days in DMEM complete medium. The culture supernatant was collected and mixed with chondrogenic induction medium at a 1:1 ratio. After BMSCs were cultured to form cell pellets, the mixed culture medium was used to culture the cell pellets for 14 days with a medium change every other day. Cell pellets were fixed, embedded, and sectioned for immunofluorescence, HE, and toluidine blue staining. The cell pellets were also subjected to qRT-PCR and WB.

Establishment of the OA mouse model

All animal husbandry and experiments were approved by the Animal Ethics Committee of Shanghai Shengchang Biotechnology Co., Ltd (2023-02-SGKYJS-CWG-042). 10-week-old C57BL/6 male mice were subjected to ACLT surgery. Briefly, after general anesthesia was induced by 3% isoflurane, the animals were subjected to aseptic surgery. The skin was incised to expose the joint cavity. After severing the anterior cruciate ligament (ACL) under a surgical microscope, the wound was flushed with PBS and sutured. The left knee was used as a sham operation group by opening the joint capsule only without severing the ACL. Four weeks after surgery, the joint cavities were injected with 10 μL PBS, PAHM, A-Lipo/PHM, or A-Lipo/PAHM every two weeks. The mice received treatment for eight weeks before being sacrificed, and specimens were harvested for further experiments.

In vivo fluorescence imaging

After injection of DiD-Lipo into the right knee and DiD-Lipo/AHM into the left knee of C57BL/6 mice, the fluorescence intensity was measured using an IVIS-spectrum system (Xenogen, USA) at 1, 1, 3, 7, 14, and 28 days.

Gait analysis

C57BL/6 mice had their left hind paw colored in red while their right hind paw was colored in blue, which were then placed at the entrance of a paper-covered channel and allowed to walk along the channel. Each mouse underwent a minimum of three times tests. The stride length they left on the paper was assessed.

X-ray and microCT

The knee specimens were placed on X-ray equipment (Faxitron X-ray, USA) to obtain radiographs and were scanned by microCT (Scanco Medical, Switzerland) at a 70 kV scanning voltage and 130 μA scanning current. The microCT images were used for 3D reconstruction of the knee joint using Mimics Medical 21.0 software. Osteometry parameters, such as BMD, BV/TV, Tb.Pf, and SBP.Th were analyzed using

CTAn software.

Histological assessment

Heart, liver, spleen, lung, kidney, and knee joint specimens of mice were collected, fixed, embedded and sectioned. All tissue sections were subjected to HE staining. Additionally, toluidine blue and safranin-O/fast green were applied to stain joint tissue sections. OARSI grades and the Mankin score were calculated.

Immunohistochemical staining

Briefly, the sections were exposed to a primary antibody at 4 °C for 12 hours and were incubated with an HRP-conjugated secondary antibody for 2 hours at RT. Last, the staining was developed with DAB. Each staining step of the sections requires at least three washes with PBS. The samples were observed, and images were obtained under a bright-field microscope.

These primary antibodies were employed： anti-p16INK4a antibody (1:50, Santa), anti-p21 antibody (1:50, Santa), anti-COL2A1 antibody (1:50, Santa), anti-MMP13 antibody (1:200, ABclonal).

Statistical analysis

All results are presented as the mean ± standard deviation (SD). The number of independent biological replicates is shown in the figure legends. Data were statistically analyzed using the two-tailed Student's t-test or one-way ANOVA with Tukey's multiple comparisons test. $p < 0.05$ was considered statistically significant. GraphPad Prism 20.0 was used for data analysis.

References

[1] T. Ito, Y. Yeo, C. B. Highley, E. Bellas, C. A. Benitez, D. S. Kohane, *Biomaterials* **2007**, *28*, 975.
[2] Y. Lei, Y. Wang, J. Shen, Z. Cai, Y. Zeng, P. Zhao, J. Liao, C. Lian, N. Hu, X. Luo, W. Cui, W. Huang, *Adv. Funct. Mater.* **2021**, *31*, 2105084.
[3] a)T. N. Snyder, K. Madhavan, M. Intrator, R. C. Dregalla, D. Park, *J Biol Eng*

2014, 8, 1; b)Q. Zhang, X. Wei, Y. Ji, L. Yin, Z. Dong, F. Chen, M. Zhong, J. Shen, Z. Liu, L. Chang, *J Mater Chem B* **2020**, 8, 5441; c)A. M. Gielen, M. Ankone, D. W. Grijpma, A. A. Poot, *Biomacromolecules* **2022**.

[4] T. Dai, C. Wang, Y. Wang, W. Xu, J. Hu, Y. Cheng, *ACS Appl. Mater. Interfaces* **2018**, *10*, 15163.

[5] X. Zhao, S. Liu, L. Yildirimer, H. Zhao, R. Ding, H. Wang, W. Cui, D. Weitz, *Adv. Funct. Mater.* **2016**, *26*, 2809.

[6] J. Weischenfeldt, B. Porse, *CSH protoc* **2008**, *2008*, pdb. prot5080.

Supplementary Figures

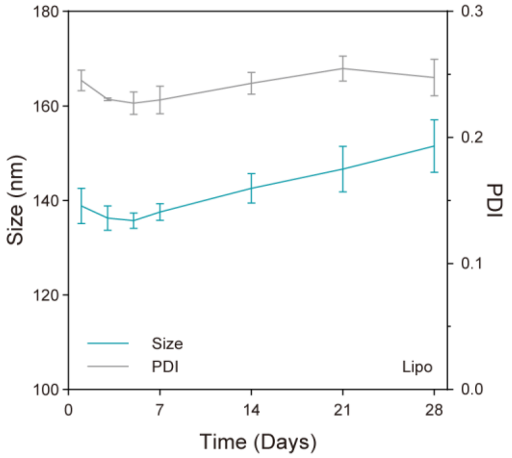

Figure S1. Stability of Lipo *in vitro* over 28 days. Data are presented as mean ± SD (n=3).

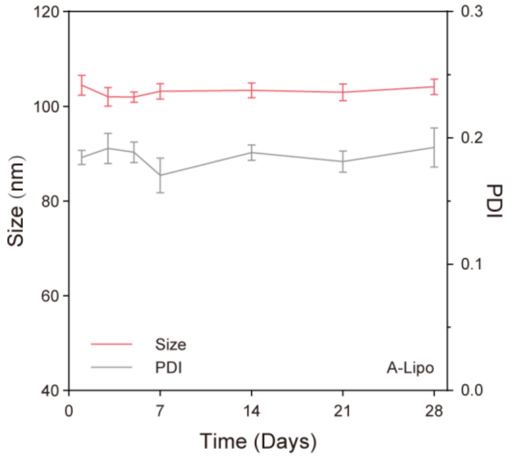

Figure S2. Stability of A-Lipo *in vitro* over 28 days. Data are presented as mean ± SD (n=3).

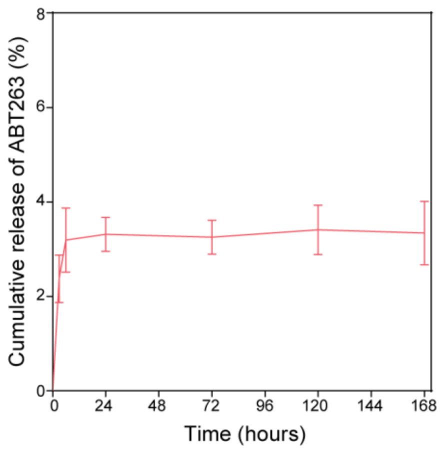

Figure S3. The cumulative release profile of ABT263 from A-Lipo in PBS containing 1% Tween 80. Data are presented as mean ± SD (n=3)

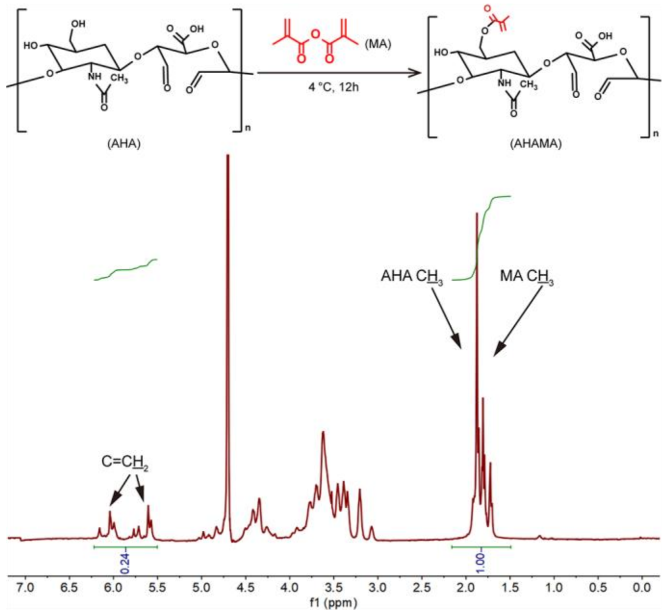

Figure S4. Schematic diagram of the chemical reaction between AHA and MA, and ^{1}H NMR spectra of MA proton peak integrals.

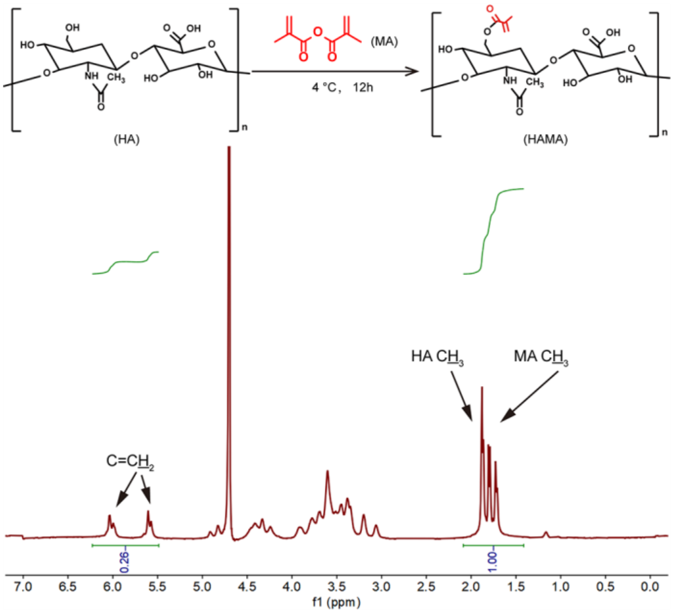

Figure S5. Schematic diagram of the chemical reaction between HA and MA, and ^{1}H NMR spectra of MA proton peak integrals.

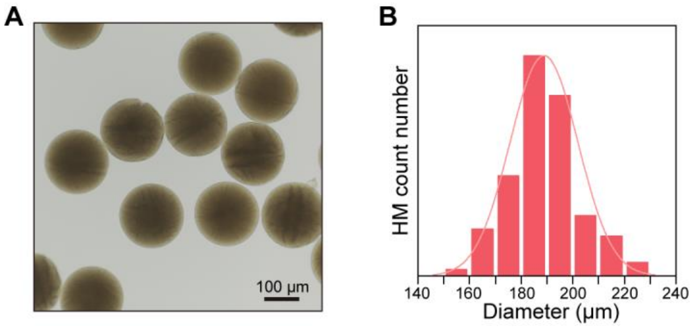

Figure S6. (A) HM images acquired with a bright-field microscope. Scale bar: 100 μm. (B) Diameter distribution of HM calculated using imageJ.

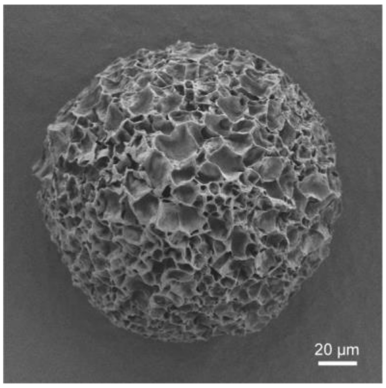

Figure S7. Representative SEM images of HM. Scale bar: 20 μm.

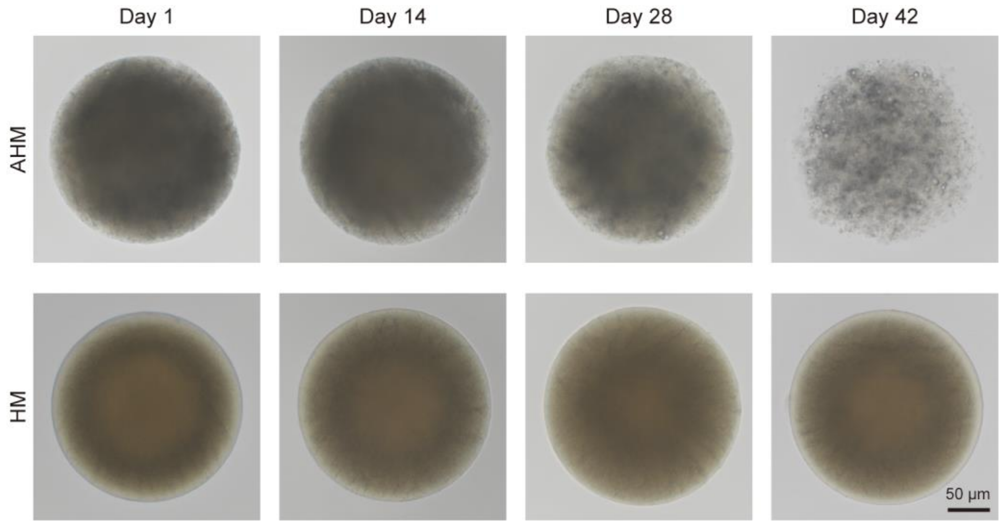

Figure S8. *In vitro* degradation properties of AHM and HM observed under bright-field microscope. Scale bar: 50 μm.

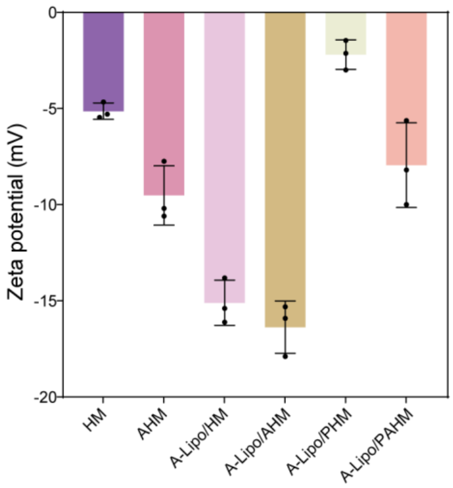

Figure S9. The Zeta potentials of HM, AHM, A-Lipo/HM, A-Lipo/AHM, A-Lipo/PHM, and A-Lipo/PAHM. Data are presented as mean ± SD (n=3).

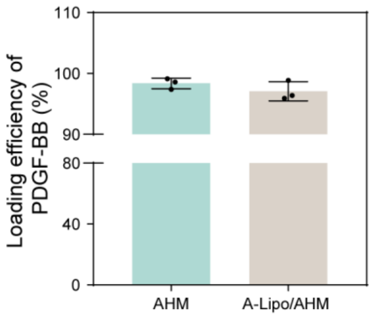

Figure S10. The PDGF-BB loading efficiency of AHM and A-Lipo/AHM. Data are presented as mean ± SD(n=3).

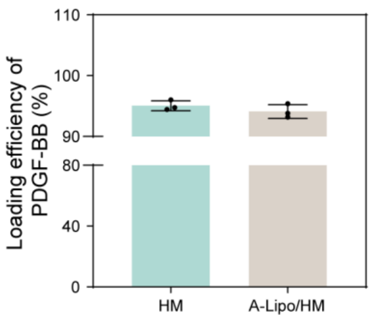

Figure S11. The PDGF-BB loading efficiency of HM and A-Lipo/HM. Data are presented as mean ± SD(n=3).

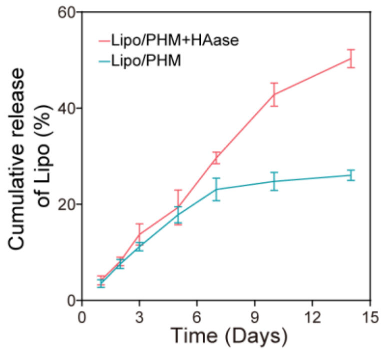

Figure S12. Measuring the cumulative release rate of Lipo within Lipo/PHM under PBS or PBS containing HAase (1500 U/mL) by quantification of the fluorescence intensity of FITC-labeled Lipo. Data are presented as mean ± SD (n=3).

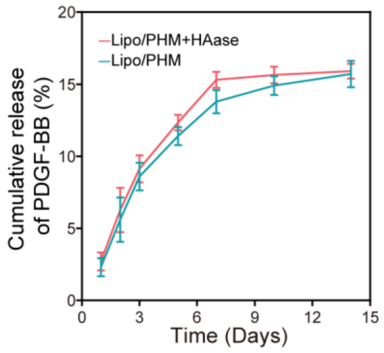

Figure S13. Measuring the cumulative release rate of PDGF-BB within Lipo/PHM under PBS or PBS containing HAase (1500 U/mL) using ELISA kit. Data are presented

as mean ± SD (n=3).

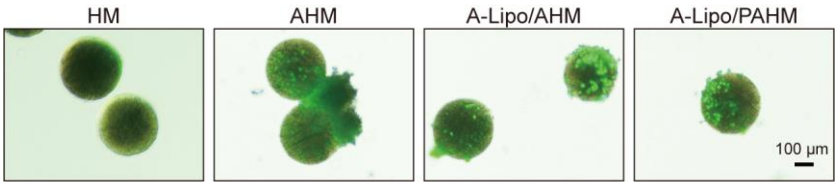

Figure S14. Representative fluorescence images of BMSCs cultured on HM, AHM, A-Lipo/AHM, and A-Lipo/PAHM. BMSCs (Stained with Calcein-AM, Green). Scale bar: 100 μm.

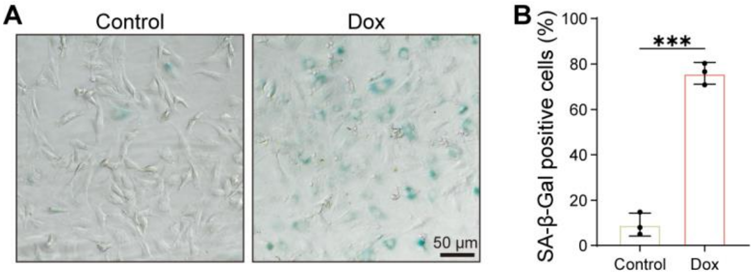

Figure S15. (A) Representative images of SA-β-Gal staining of chondrocytes. (B) Quantification of SA-β-Gal-positive cells after 14 days of Dox intervention (n=3). scale bar: 50μm. Data are presented as mean ± SD. Student's two-tailed t test was used for data analysis. ***$P < 0.001$.

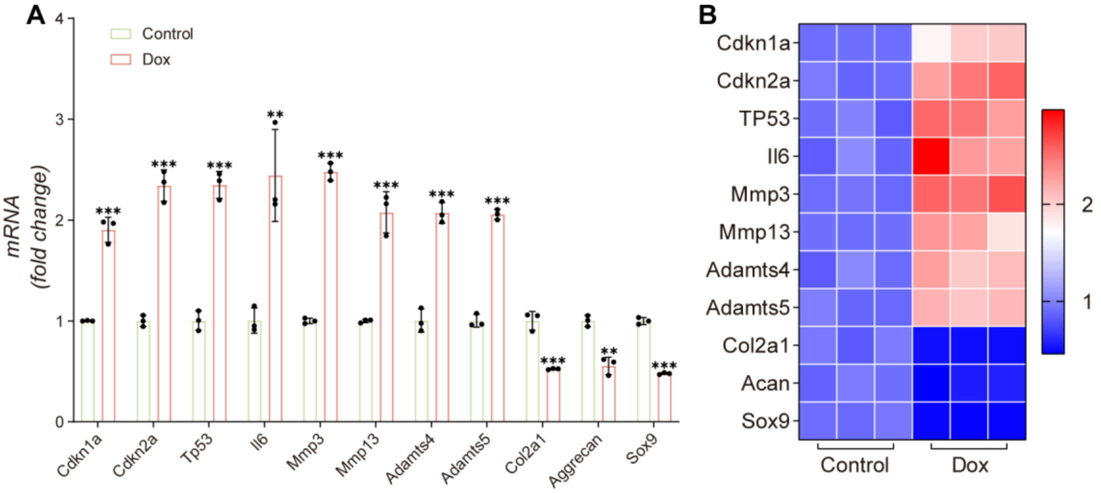

Figure S16. (A-B) Quantitative analysis and heatmap of mRNA expression of *Cdkn1a, Cdkn2a, Tp53, Il6, Mmp3, Mmp13, Adamts4, Adamts5, Col2a1, Aggrecan, and Sox9* in chondrocytes after 14 days of Dox intervention. (n=3). Data are presented as mean ±

SD. Student's two-tailed t test was used for data analysis. $^{**}p < 0.01$, $^{***}p < 0.001$.

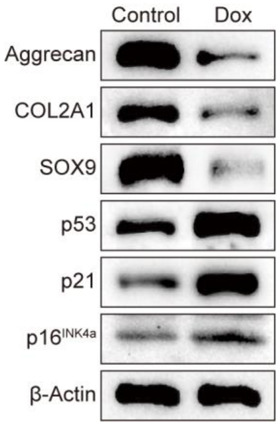

Figure S17. Western blotting analysis of Aggrecan, COL2A1, SOX9, p53, p21, and p16^{INK4a} after 14 days of Dox intervention.

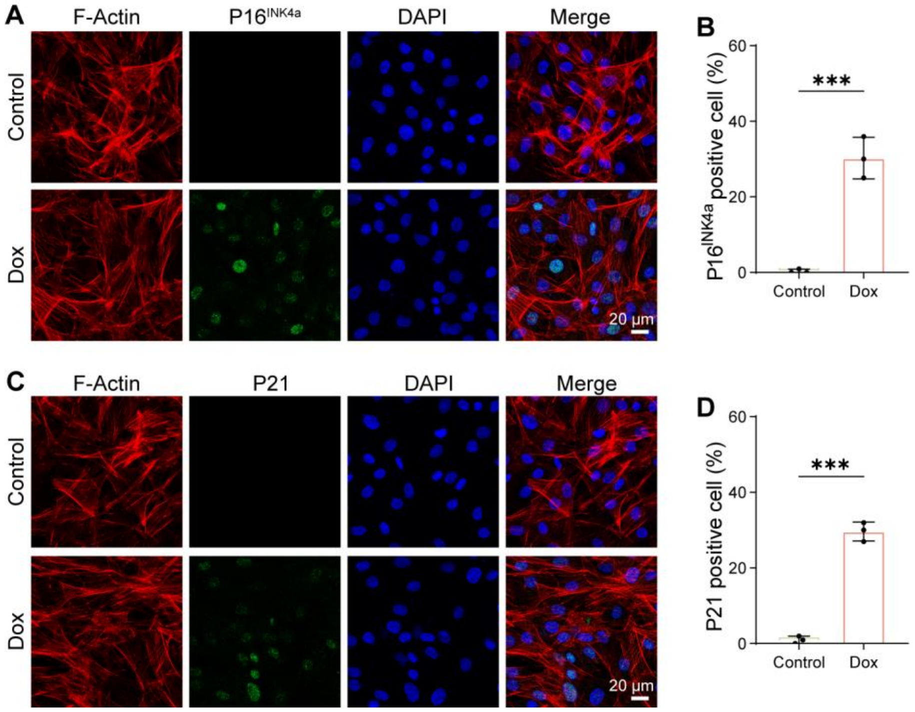

Figure S18. (A-B) Representative immunofluorescence images of P16^{INK4a} and the percentage of positive cells of P16^{INK4a} (n=3). Green (P16^{INK4a}), Red (F-Actin)， Blue (DAPI). Scale bar: 20 µm. c-d) Representative immunofluorescence images of P21 and the percentage of positive cells of P21 (n=3). Green (P21), Red (F-Actin)， Blue (DAPI). Scale bar: 20 µm. Data are presented as mean ± SD. Student's two-tailed t test

was used for data analysis. ***$P < 0.001$.

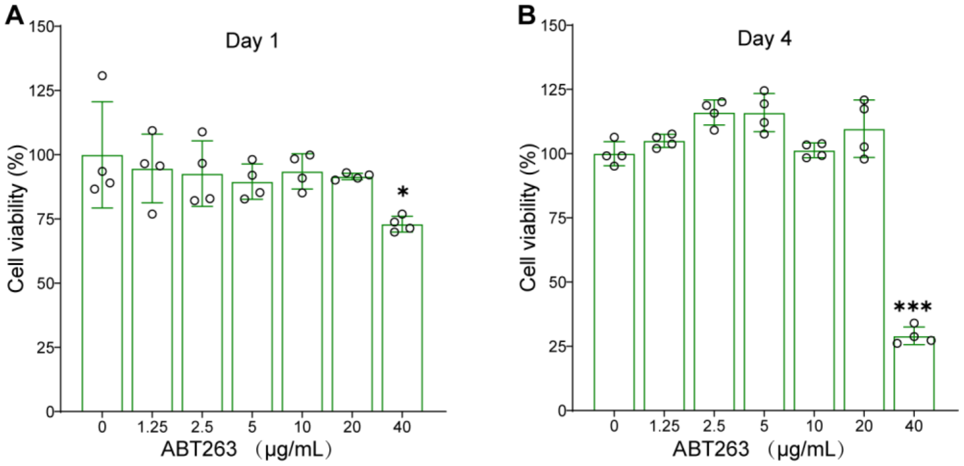

Figure S19. (A-B) Cell viability analysis of chondrocytes after treatment with ABT263 (1-40 μg/mL) for 1 day or 4 day (n=4). Data are presented as mean ± SD. Student's two-tailed t test was used for data analysis. *$P < 0.05$, ***$P < 0.001$.

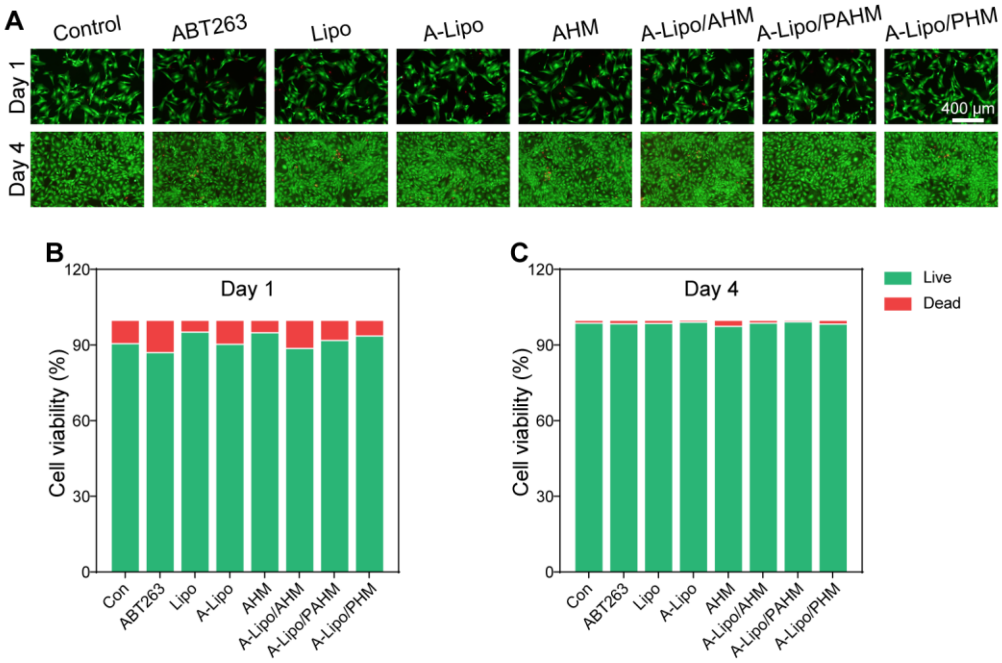

Figure S20. (A) Representative images of Live/dead cell staining of chondrocytes with different treatments for 1 day or 4 day. Green (Calcein-AM), Red (PI). Scale bar: 400 μm. (B-C) Quantitative analysis of cell viability (n=3).

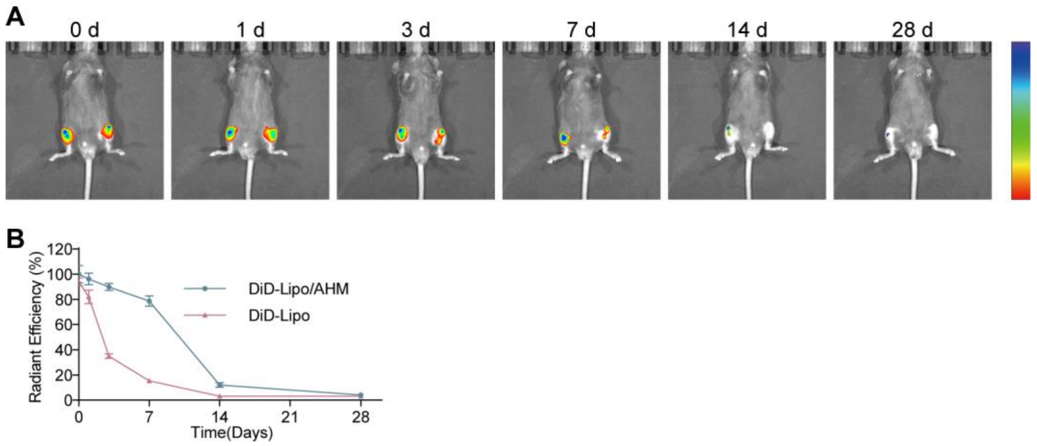

Figure S21. (A) IVIS images at different time points *in vivo*. DiD-Lipo (left), DiD-Lipo/AHM (right). (B) Relative radiant efficiency of IVIS images at different time points (n=3).

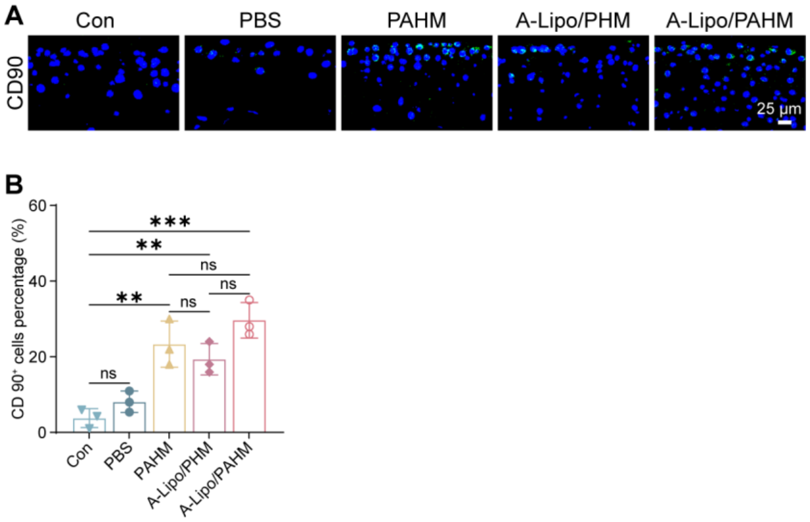

Figure S22. Assessment of the ability of microspheres to recruit stem cells *in vivo*. (A) images of IF staining of CD90, Green (Stem Cell Marker:CD90), blue (DAPI). Scale bar: 25μm. (B) Percentage of CD90 positive cells (n=3). Data are presented as mean ± SD. One-way ANOVA and Tukey's multiple-comparisons test were used for data analysis. ns: no significance, *$P < 0.05$, **$P < 0.01$, ***$P < 0.001$.

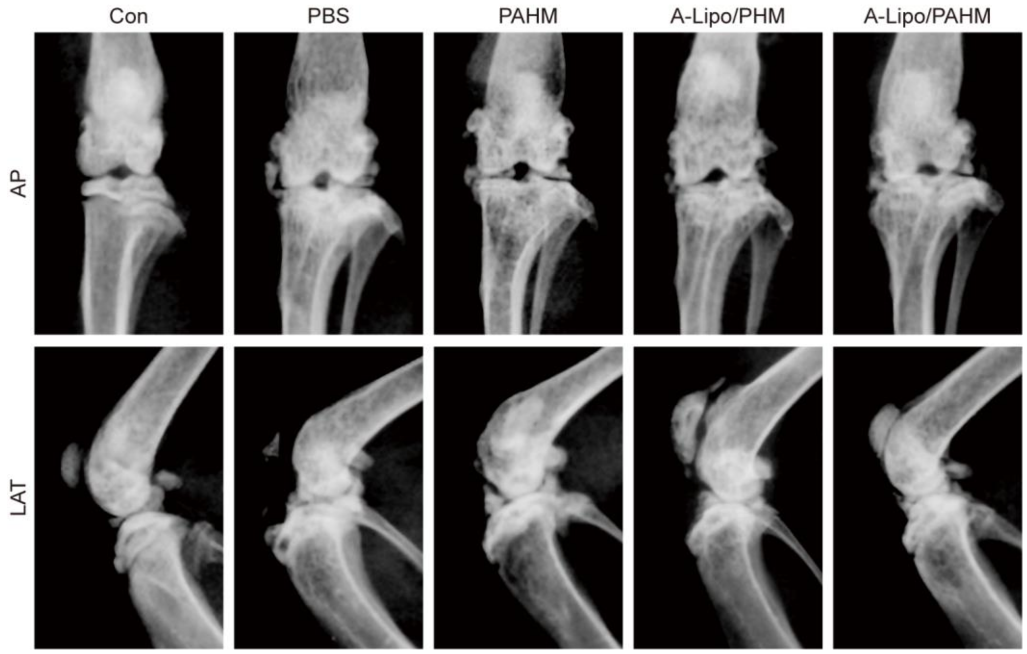

Figure S23. Representative X-ray films of the mice knee joints in anterior-posterior (AP) and lateral (LAT).

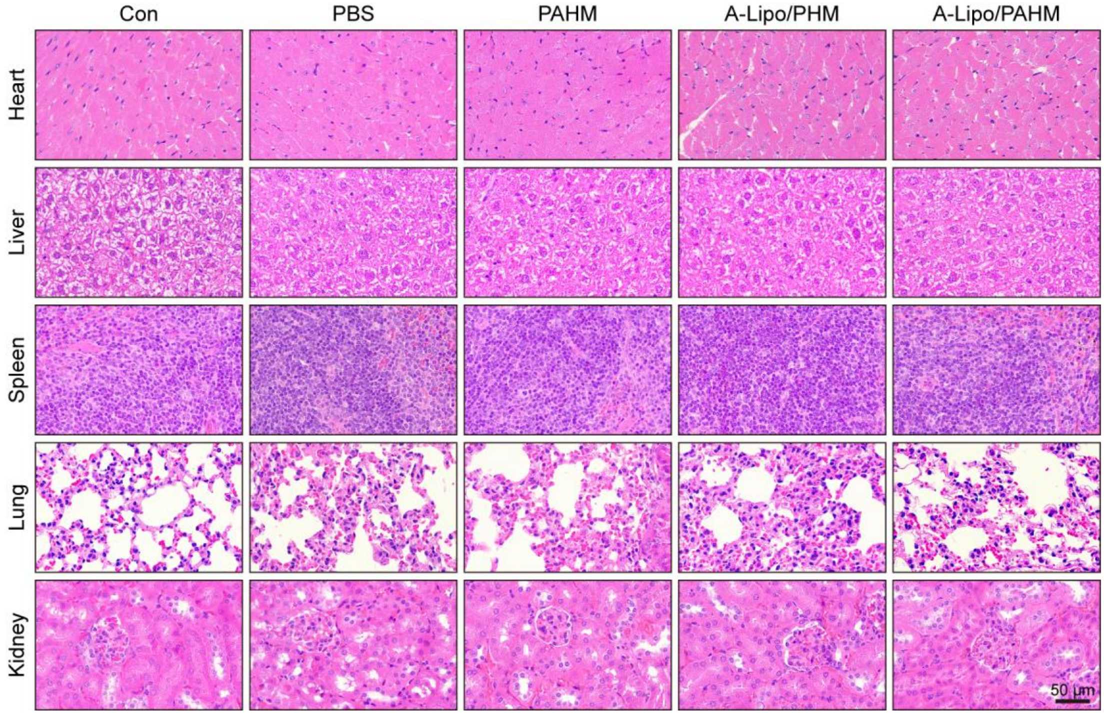

Figure S24. To assess the biocompatibility of microspheres *in vivo*. Representative images of H&E staining of heart, liver, spleen, lung, and kidney. Scale bar: 50 µm.

Table S1. List of primers for qRT-PCR

Gene	Species	Category	Primer (5'-3')

Cdkn1a	Mouse	Forward	CCTTGTCGCTGTCTTGCACTCTG
		Reverse	GCTGGTCTGCCTCCGTTTTCG
Cdkn2a	Mouse	Forward	CAAGAGCGGGGACATCAAGACATC
		Reverse	CACAAAGACCACCCAGCGGAAC
Tp53	Mouse	Forward	CGACATACTCAGCACCAGCATCAC
		Reverse	GCAGCGTCTCACGACCTCAG
Il6	Mouse	Forward	CTTCTTGGGACTGATGCTGGTGAC
		Reverse	TCTGTTGGGAGTGGTATCCTCTGTG
Mmp3	Mouse	Forward	GACGATGATGAACGATGGACAGAGG
		Reverse	TGTGGAGGACTTGTAGACTGGGTAC
Mmp13	Mouse	Forward	ACAGTTGACAGGCTCCGAGAAATG
		Reverse	CCACATCAGGCACTCCACATCTTG
Adamts4	Mouse	Forward	AGCCACAGCAGCCTCAGAGAC
		Reverse	GTTGCCAGCCACCAGGACTTG
Adamts5	Mouse	Forward	TCCTCTTGGTGGCTGACTCGTC
		Reverse	AGGCGGATGTGGTTCTCAATGC
Col2a1	Mouse	Forward	GGTGGAGCAGCAAGAGCAAGG
		Reverse	TCAGTGGACAGTAGACGGAGGAAAG
Aggrecan	Mouse	Forward	GGAGACCCAGACAGCAGAAACAAC
		Reverse	GCAGGTGGCTCCATTCAGACAAG
Sox9	Mouse	Forward	CACTACAGCGAGCAGCAGCAG
		Reverse	CGTATTGCGAGCGGGTGATGG
Il1β	Mouse	Forward	CACTACAGGCTCCGAGATGAACAAC
		Reverse	TGTCGTTGCTTGGTTCTCCTTGTAC
iNOS	Mouse	Forward	CACCACCCTCCTCGTTC
		Reverse	CAATCCACAACTCGCTCC
Arg1	Mouse	Forward	ATATCTGCCAAAGACATCG
		Reverse	TCACCTTGCCAATCCC
Il10	Mouse	Forward	GGCCCAGAAATCAAGGAGCA
		Reverse	GCCTTGTAGACACCTTGGTCTT
Gapdh	Mouse	Forward	ACGGCAAGTTCAACGGCACAG
		Reverse	CGACATACTCAGCACCAGCATCAC
